# Dataset on the abundance, enrichment and partitioning of chemical elements between the filtered, particulate and sedimentary phases in the Cai River estuary (South China Sea)

**DOI:** 10.1016/j.dib.2021.107412

**Published:** 2021-09-23

**Authors:** Sofia E. Koukina, Nikolay V. Lobus, Alexander V. Shatravin

**Affiliations:** a Shirshov Institute of Oceanology, Russian Academy of Sciences, Nahimovskiy pr. 36, Moscow 117997, Russia; b Timiryazev Institute of Plant Physiology, Russian Academy of Sciences, Botanicheskaya st. 35, Moscow 127276, Russia; c Prokhorov General Physics Institute of the Russian Academy of Sciences, Vavilova st. 38, Moscow 119991, Russia

**Keywords:** Organic geochemistry, Major elements, Trace elements, Rare earth elements, Enrichment factor, Geoaccumulation index, Partitioning coefficient

## Abstract

This data article refers to the paper entitled “Multi-element signatures in solid and solution phases in a tropical mixing zone: A case study in the Cai River estuary, Vietnam” (Koukina et al., 2021), which considers the fate of major, trace, and rare-earth elements transported through the estuarine geochemical filter of the typical tropical estuary. The present work contributes to the local geochemical baselines as a background for long-term monitoring of potential hazardous elements. Therefore, the dataset covers the abundance, enrichment, and partitioning parameters of 53 chemical elements in the water, suspended particulate matter, and bottom sediment samples collected in the Cai River estuary and the adjacent part of the Nha Trang Bay (South China Sea) between July and August 2013. The total filtered, particulate, and sedimentary elements were determined by atomic emission and inductively coupled plasma mass spectrometry (ICP-AES; ICP-MS). The environmental indices (the enrichment factor and geoaccumulation index) and partition coefficients were calculated from the total element contents. The data provided is essential for the comprehensive environmental assessment of the anthropogenic impact on the coastal ecosystem as well as for the evaluation and modelling of element fractionation and mobility at the estuarine gradients.

## Specifications Table

**Table:** 

Subject	Environmental Chemistry
Specific subject area	The Environmental Geochemistry of Estuaries, Aquatic Geochemistry, Geochemical cycles of elements, Ocean Ecology
Type of data	Tables, Graphs, Figures
How data were acquired	TOC 5000-V-CPH analyzer (Shimudzu Co., Japan), ICP-AES (ICAP-61, Thermo Jarrell Ash, USA), ICP-MS (X-7, Thermo Elemental, USA), total dissolution in HNO_3_ + HClO_4_ (3:1 by volume, Merck) in an autoclave system (Аnkon-АТ-2, Russia), Microsoft Excel 2010, MATLAB R2018a (MathWorks, Inc., USA)
Data format	Raw, Analyzed
Parameters for data collection	The surface water (eight locations, sts. 1–8) and surface sediment samples (seven locations, sts. 2–8) were collected in the Cai River estuary and Nha Trang Bay during the dry season in July-August 2013. The surface water samples were collected using a plastic Niskin Bottle. The suspended particulate matter (SPM) samples were obtained by filtering water samples in an all-glass filtering system. The surface sediment samples were collected by scuba divers with a manual plastic piston corer. All sampling, sampling transportation, and preparation procedures were performed using standard clean techniques.
Description of data collection	Organic geochemistry parameters in the filtered water, SPM and sediment samples (Dissolved organic carbon (DOC), Particulate organic carbon (POC), Total dissolved nitrogen (TDN), Total carbon (TC) and Total inorganic carbon (TIC)) were determined with the analyser TOC 5000-V-CPH (Shimudzu Co., Japan). Standard methods (ICP-AES and ICP-MS) were applied to determine the content of the chemical elements. The elemental analysis of the filtered water samples and the solution obtained by the total dissolution of SPM and sediment samples was determined with the ICAP -61 (Thermo Jarrell Ash, USA) with the X-7 (Thermo Elemental, USA).
Data source location	The latitude and longitude of the sampling sites are given in Table 1. Preparation of the samples and data analysis were conducted at the Shirshov Institute of Oceanology, Russian Academy of Sciences, Moscow, Russia. The analytical procedure was carried out at the Institute of Microelectronics Technology and High Purity Materials, Russian Academy of Sciences, Chernogolovka, Moscow Region, Russia.
Data accessibility	With the article and available on a public repository. Repository name: Mendeley Data Data identification number: 10.17632/t636fc98vt.1 Direct URL to data: https://data.mendeley.com/datasets/t636fc98vt/1
Related research article	Koukina S.E., Lobus N.V., Shatravin A.V. Multi-element signatures in solid and solution phases in a tropical mixing zone: A case study in the Cai River estuary, Vietnam // Chemosphere. 2021. V. 280. 130951. https://doi.org/10.1016/j.chemosphere.2021.130951

## Value of the Data

• The data on element enrichment and partitioning in the solid and solution phases of the typical tropical estuary is essential for an adequate assessment of hazardous elements, their possible pathways, and the potential risk to the coastal environment under multiple pressures.
• The data is useful for environmental scientists as well as for decision makers in order to prevent chemical pollution and implement sustainable development goals in the coastal Vietnam.
• The data may be used for the evaluation of the local geochemical background and further environmental monitoring and assessment of the developing Nha Trang Bay region as well as for the global modelling of element fractionation and mobility at the estuarine gradients.

## 1 Data Description

**Fig. 1:**
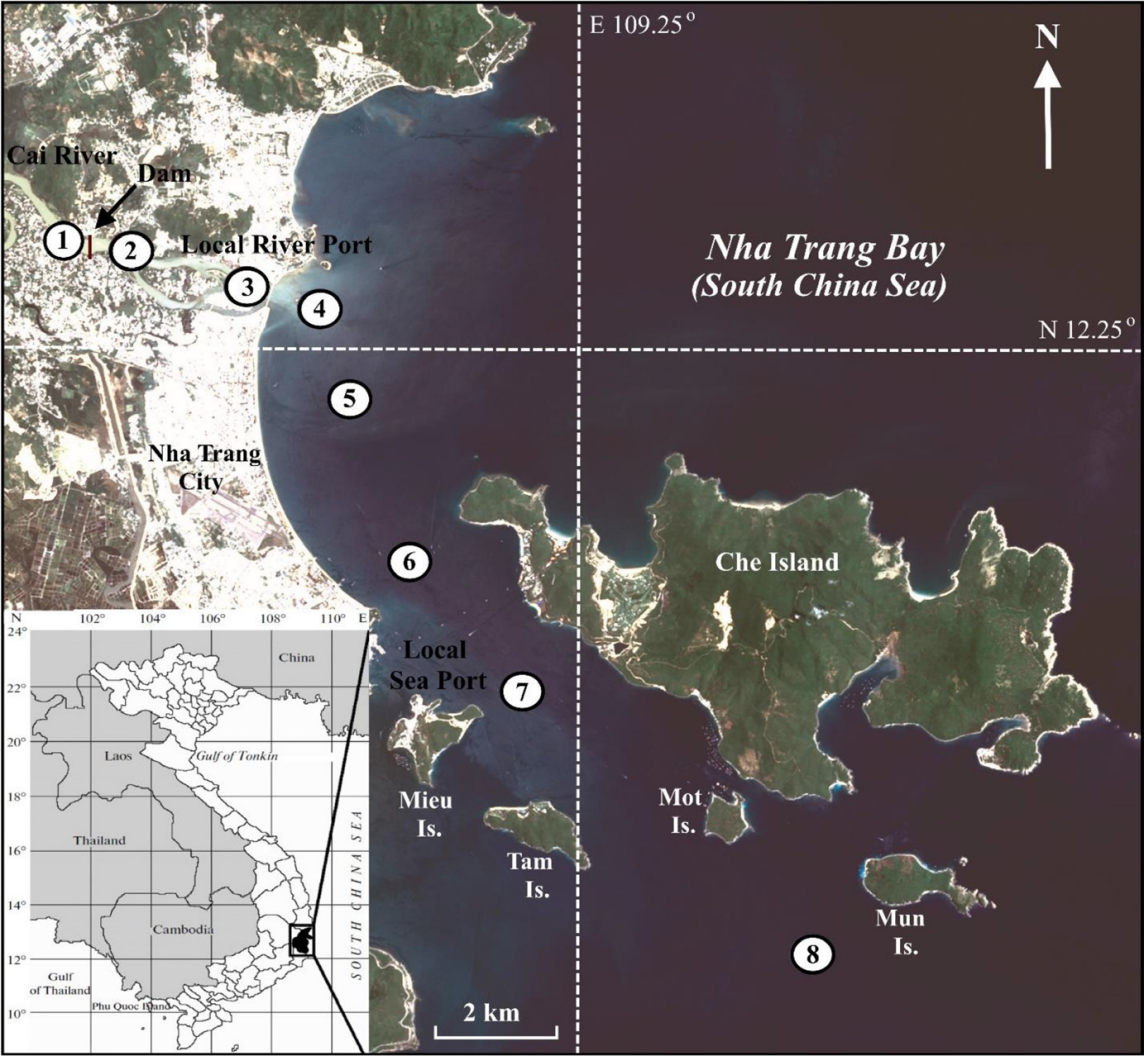
Sampling site locations (Landsat 8 satellite image, 16 August 2013) [1].

Table 1 and Fig. 1 show the latitude and longitude of the sampling sites.

**Table 1:** Location of the sampling sites.

Station	Longitude, N	Latitude, E
1	12.271	109.167
2	12.268	109.175
3	12.262	109.197
4	12.261	109.204
5	12.246	109.208
6	12.219	109.225
7	12.198	109.242
8	12.152	109.293

Table 2 reports on the detection limits and measured and certified values of element concentrations in the Standard Reference Material (“Trace Metals in Drinking Water” (EU)) that were applied for the evaluation of the precision and validity of the elemental analysis of the filtered water samples. Table 3 reports on the detection limits, measured, and certified values of element concentrations in the Standard Reference Materials (Andesite, AGV-2, and Essexite Gabbro SRM-2A) that were applied for the evaluation of the precision and validity of the elemental analysis of the SPM and sediment samples.

**Table 2:** Detection limits and measured and certified values of element concentrations in Standard Reference Material.

	Detection limit				“Trace Metals in Drinking Water”(CRM-TMDW-A-250)^4^	
Element	Fresh water (< 0.01 ‰)	Brackish water (1-10 ‰)^1^	Brackish water (10-20 ‰)^2^	Sea water (> 20 ‰)^3^	Measured value	Certifiedvalue ± SD
**Major elements, mg L^−1^**						

Na	0.017	0.017	0.017	0.345	5.872	5.936 ± 0.017
Mg	0.008	0.008	0.008	0.153	8.761	8.956 ± 0.018
K	0.01	0.01	0.01	0.193	2.429	2.488 ± 0.009
Ca	0.018	0.018	0.018	0.37	35.06	35.19 ± 0.07

**Trace elements, µg L^–1^**						

Li	0.003	0.017	0.034	0.137	20.31	20.23 ± 0.12
Be	0.002	0.008	0.016	0.063	20.41	20.33 ± 0.12
B	0.6	3	2	26	< d/l	n.d.
Al	1	3	4	53	121.9	120.0 ± 0.1
Ti	0.7	4	7	28	< d/l	n.d.
V	0.07	0.4	1	3	30.01	30.06 ± 0.02
Cr	0.6	3	6	24	20.24	20.02 ± 0.01
Mn	0.04	0.2	0.4	2	39.77	39.6 ± 0.1
Fe	6	6	15	115	98.99	100.1 ± 0.1
Co	0.08	0.4	1	3	25.34	25.03 ± 0.02
Ni	0.3	0.8	2	7	61.71	60.5 ± 0.5
Cu	0.3	0.6	3	12	20.46	20.03 ± 0.01
Zn	0.4	0.9	4	18	70.73	70.5 ± 0.5
As	0.05	0.2	0.5	2	80.21	80.4 ± 1.0
Se	0.3	1	3	10	10.19	10.13 ± 0.16
Br	8	40	81	323	< d/l	n.d.
Rb	0.006	0.03	0.06	0.24	10.35	10.09 ± 0.07
Zr	0.007	0.033	0.065	0.261	< d/l	n.d.
Nb	0.004	0.022	0.044	0.174	< d/l	n.d.
Sr	0.06	0.3	1	2	246.4	250.3 ± 0.2
Mo	0.016	0.079	0.158	0.632	101.2	99.0 ± 0.7
Ag	0.003	0.017	0.034	0.138	1.99	1.97 ± 0.01
Cd	0.006	0.028	0.055	0.221	10.34	10.01 ± 0.01
Sn	0.012	0.061	0.121	0.485	< d/l	n.d.
Sb	0.008	0.04	0.08	0.323	10.46	9.96 ± 0.14
Te	0.005	0.027	0.054	0.215	2.98	2.95 ± 0.04
Cs	0.005	0.024	0.047	0.189	< d/l	n.d.
Ba	0.03	0.1	0.3	1	49.21	50.7 ± 0.8
W	0.018	0.088	0.176	0.705	< d/l	n.d.
Tl	0.0004	0.002	0.004	0.016	10.0	9.8 ± 0.16
Pb	0.02	0.08	0.2	0.6	39.98	39.3 ± 0.2
Bi	0.0008	0.004	0.004	0.034	9.71	10.01 ± 0.01
Th	0.002	0.011	0.022	0.086	< d/l	n.d.
U	0.0006	0.003	0.006	0.023	10.11	10.01 ± 0.01

**Rare-earth elements, ng L^–1^**						

Sc	60	300	1000	2000	< d/l	n.d.
Y	1	7	14	57	< d/l	n.d.
La	3	13	26	105	< d/l	n.d.
Ce	2	9	17	68	< d/l	n.d.
Pr	0.4	2	4	15	< d/l	n.d.
Nd	1	5	11	42	< d/l	n.d.
Sm	0.7	3	7	28	< d/l	n.d.
Eu	0.4	2	4	15	< d/l	n.d.
Gd	0.5	3	5	20	< d/l	n.d.
Tb	0.2	1	2	8	< d/l	n.d.
Dy	0.7	4	7	30	< d/l	n.d.
Ho	0.1	0.7	1	5	< d/l	n.d.
Er	0.6	3	6	25	< d/l	n.d.
Tm	0.2	0.8	2	6	< d/l	n.d.
Yb	0.3	1	3	10	< d/l	n.d.
Lu	0.1	0.4	1	3	< d/l	n.d.

*Note:* n.d. – no data, < d/l – below detection limit; 1 –5 times diluted (Milli-Q); 2 – dilution 10 times; 3 – dilution 40 times; 4 – Matrix: 2% HNO_3_ and 0.01% HF.

**Table 3:** Detection limits and measured and certified values of element concentrations in Standard Reference Materials.

		Andesite, AGV – 2 (United States Geological Survey)		Essexite Gabbro – 1A (Russian Geochemical Standard, SRM-521-84P)		Black shale SLg – 1 (Russian Geochemical Standard, SRM-8550-04)	
Element	Detectionlimit	Measured value	Certified value ± SD	Measured values	Certified value ± SD	Measured values	Certified value ± SD
**Major elements, %**							

Na	0.003	2.75	3.11 ± 0.09	2.08	2.09 ± 0.03	0.98	0.94 ± 0.1
Mg	0.002	1.07	1.08 ± 0.02	4.46	4.22 ± 0.06	−	1.85 ± 0.05
Al	0.002	6.82	8.95 ± 0.11	7.83	7.87 ± 0.04	8.6	8.2 ± 0.16
P	0.004	0.21	0.21 ± 0.01	0.46	0.44 ± 0.01	0.05	0.05 ± 0.004
K	0.003	2.16	2.39 ± 0.09	2.41	2.46 ± 0.041	2.49	2.37 ± 0.09
Ca	0.005	3.43	3.72 ± 0.09	7.79	7.84 ± 0.06	0.8	0.79 ± 0.07
Ti	0.0001	0.58	0.63 ± 0.13	1.0	1.03 ± 0.02	0.52	0.53 ± 0.02
Mn	0.0002	0.062	0.077 ± 0.002	0.12	0.13 ± 0.008	0.085	0.085 ± 0.001
Fe	0.003	4.06	4.68 ± 0.09	8.18	8.16 ± 0.17	5.32	5.23 ± 0.12

**Trace elements, µg g^–1^**							

Li	0.04	8.81	11*	14.2	14 ± 3	55.3	50 ± 8
Be	0.01	2.1	2.3 ± 0.4	2.2	2.0 ±	2.0	2.4*
V	2	130	120 ± 5	249	240 ± 20	135	122 ± 15
Cr	1	15.6	17 ± 2	51.7	55 ± 4	122	116 ± 8
Co	0.2	16.8	16 ± 1	45.0	40 ± 5	19.3	20 ± 3
Ni	1	18.6	19 ± 3	38.1	50 ± 5	54.2	50 ± 7
Cu	1	48.5	53 ± 4	61.2	68 ± 7	37.2	39 ± 7
Zn	1	81.0	86 ± 8	122.3	120 ± 10	81.9	97 ± 13
Ga	0.1	22.2	20 ± 1	22	19 ± 2	18.8	18 ± 3
As	0.1	0.5	n.d.	1.8	1.8 ± 0.2	26.3	46 ± 8
Rb	0.1	77.1	68.6 ± 2.3	88.5	73 ± 4	114	112 ± 11
Sr	0.1	672	658 ± 17	2454	2300 ± 200	146	142 ± 15
Zr	0.1	255	230 ± 4	284	240 ± 20	186	176 ± 16
Nb	0.05	14.1	15 ± 1	10.1	8 ± 1	11.4	12 ± 2
Mo	0.08	2.0	n.d.	1.2	1.5 ± 0.5	0.84	0.8*
Ag	0.07	0.091	n.d.	0.11	0.10 ± 0.05	0.5	0.47 ± 0.08
Cd	0.06	0.086	n.d.	< d/l	n.d.	0.3	0.4*
Sn	0.3	2.7	2.3 ± 0.4*	2.7	3.7 ± 0.6*	3	3.2*
Sb	0.05	0.51	0.6*	0.31	n.d.	1.2	1*
Cs	0.01	1.2	1.16 ± 0.08*	3.9	3.8 ± 0.4	4.21	4.0 ± 0.7
Ba	0.08	1160	1140 ± 32	1427	1300 ± 100	378	376 ± 46
Tl	0.003	0.32	0.27*	0.17	n.d.	0.61	n.d.
Pb	0.1	14.0	13 ± 1	20.6	17 ± 2	15.8	15 ± 3
Bi	0.006	0.043	n.d.	0.047	n.d.	0.11	n.d.
Th	0.03	6.6	6.1 ± 0.6	10.1	9 ± 1	8	7.1 ± 1.1
U	0.009	1.9	1.88 ± 0.16	2.8	2.0 ± 0.5	1.7	1.7 ± 0.2

**Rare-earth elements, µg g^–1^**							

Sc	0.1	12.2	13 ± 1	21.8	27 ± 3	22.5	20 ± 3
Y	0.01	20.0	20 ± 1	33.7	30 ± 4	26	26 ± 4
La	0.02	41.7	38 ± 1	90.7	80 ± 20	28.6	28 ± 5
Ce	0.03	76.1	68 ± 3	204	150 ± 10	59.1	53 ± 8
Pr	0.01	8.4	8.3 ± 0.6	26	15 ± 5	7	6 ± 1
Nd	0.01	33.1	30 ± 2	100	70 ± 10	27.6	25 ± 4
Sm	0.01	5.8	5.7 ± 0.3*	17.6	17 ± 1	5.6	5.4 ± 0.8
Eu	0.01	1.6	1.54 ± 0.1*	4.0	5 ± 1	1.3	1.2 ± 0.2
Gd	0.01	4.9	4.69 ± 0.26	12.3	10 ± 3	5.1	4.5 ± 0.8
Tb	0.003	0.69	0.64 ± 0.04	1.5	1.4 ± 0.2	0.77	0.74*
Dy	0.009	3.8	3.6 ± 0.2	6.9	6 ± 1	4.7	4.4*
Ho	0.007	0.70	0.71 ± 0.08*	1.2	1.2 ± 0.3	0.91	0.92*
Er	0.003	2.0	1.79 ± 0.11*	2.9	3.2 ± 0.7	2.8	2.4*
Tm	0.003	0.27	0.26 ± 0.02	0.36	0.5 ± 0.2	0.37	0.33*
Yb	0.01	1.8	1.6 ± 0.20	2.2	2.9 ± 0.5	2.8	2.7 ± 0.4
Lu	0.01	0.26	0.25 ± 0.01*	0.32	0.3*	0.41	0.4 ± 0.07

⁎ Information values

Table 4 shows the distribution of the surface water layer characteristics (Salinity, total suspended sediment (TSS), DOC, TDN, DOC to TDN ratio (C/N), POC, and POC to DOC ratio (POC/DOC)) along the salinity gradient of the Cai River estuary.

**Table 4:** Organic geochemistry parameters of surface water layer.

	Stations							
	1	2	3	4	5	6	7	8
Salinity, ‰	< 0.01	3.32	8.49	15.82	24.65	32.85	33.12	33.56
TSS, mg L^−1^	50.75	41.84	33.12	11.41	4.72	1.35	1.07	1.56
POC, mg L^−1^	1.18	1.47	1.25	0.94	0.27	0.18	0.18	0.21
DOC, mg L^−1^	2.42	2.81	2.47	2.33	8.51	4.91	1.22	1.15
DTN, mg L^−1^	0.48	0.55	0.51	0.49	0.36	0.13	0.11	0.13
DOC/DTN	5.04	5.11	4.84	4.76	23.64	37.77	11.09	8.85
POC/DOC	2.05	1.91	1.98	2.48	31.52	27.28	6.78	5.48
POC, %	2.33	3.51	3.77	8.24	5.72	13.33	16.82	13.46

Table 5 reports on the distribution and mean values of the enrichment factor (EF_Al_) calculated by the double normalization of bulk element to Al in surface SPM along the salinity gradient. Table 6 reports on the distribution and mean values of the enrichment factor (EF_Fe_) calculated by the double normalization of bulk element to Fe in surface SPM along the salinity gradient. Table 7 reports on the distribution and mean values of the enrichment factor (EF_Al_) calculated by the double normalization of bulk element to Al in surface bottom sediments along the salinity gradient. Table 8 reports on the distribution and mean values of the enrichment factor (EF_Fe_) calculated by the double normalization of bulk element to Fe in surface bottom sediments along the salinity gradient.

**Table 5:** The enrichment factor (EF_Al_) of elements in surface SPM.

	Stations								
Element	1	2	3	4	5	6	7	8	Mean ± SD
**Major elements**									

Mg	0.17	0.21	0.22	0.28	0.64	11.49	13.18	20.94	5.89 ± 8.17
P	0.24	0.28	0.31	0.42	0.61	4.41	4.99	6.20	2.18 ± 2.55
K	0.34	0.37	0.40	0.48	0.73	5.89	6.39	9.29	2.99 ± 1.37
Ca	0.07	0.05	0.03	0.04	0.21	1.85	2.07	3.40	0.96 ± 0.49
Ti	0.23	0.22	0.19	0.16	0.20	0.49	0.41	0.47	0.3 ± 0.14
Mn	0.25	0.17	0.14	0.12	0.21	1.52	1.84	3.19	0.93 ± 1.14
Fe	0.46	0.46	0.46	0.55	0.75	1.06	0.52	0.48	0.59 ± 0.21

**Trace elements**									

Li	3.88	4.05	4.01	4.03	3.45	7.48	7.49	11.42	5.73 ± 2.83
Sc	0.50	0.49	0.48	0.53	0.60	2.92	2.08	2.17	1.22 ± 1.0
V	0.42	0.43	0.48	0.59	0.89	3.43	3.30	1.51	1.38 ± 1.28
Cr	0.83	0.44	0.25	0.33	0.51	3.43	3.41	7.91	2.14 ± 2.69
Co	4.50	3.20	0.34	0.32	0.49	8.37	18.14	58.19	11.7 ± 19.72
Ni	2.98	2.23	0.40	0.49	0.71	7.44	10.76	25.73	6.34 ± 8.67
Cu	1.53	0.88	0.24	0.27	0.36	8.52	10.52	29.66	6.5 ± 10.2
Zn	0.32	0.32	0.33	0.30	0.36	1.00	0.90	1.93	0.68 ± 0.58
Ga	1.22	1.17	1.15	1.17	1.07	2.79	1.03	1.29	1.36 ± 0.58
As	0.67	0.62	0.54	0.65	1.11	5.19	1.22	27.64	4.71 ± 9.4
Rb	0.80	0.78	0.77	0.77	0.90	1.69	0.86	1.16	0.97 ± 0.32
Sr	0.14	0.14	0.14	0.20	0.75	6.32	7.23	12.06	3.37 ± 4.59
Zr	0.10	0.10	0.08	0.08	0.11	0.48	0.18	0.18	0.16 ± 0.13
Nb	0.39	0.36	0.21	0.20	0.26	1.33	0.83	0.97	0.57 ± 0.42
Mo	9.99	8.81	1.73	1.89	2.52	24.99	24.68	50.98	15.7 ± 17.12
Cd	0.11	0.08	0.06	0.01	0.05	0.48	0.00	0.00	0.1 ± 0.16
Sn	1.16	1.02	1.06	0.86	1.14	5.76	3.72	4.33	2.38 ± 1.93
Sb	0.26	0.25	0.24	0.26	0.36	1.85	0.67	1.04	0.62 ± 0.57
Cs	1.50	1.40	1.37	1.13	1.27	2.85	1.04	1.37	1.49 ± 0.57
Ba	0.32	0.28	0.22	0.28	0.42	1.34	0.37	3.39	0.83 ± 1.1
Hf	0.23	0.15	0.14	0.15	0.19	0.69	0.42	0.46	0.3 ± 0.2
W	3.48	2.32	1.60	1.63	2.18	10.20	5.52	5.99	4.12 ± 2.99
Tl	1.06	1.04	0.98	0.99	1.24	1.06	1.09	1.75	1.15 ± 0.26
Pb	0.62	0.61	0.58	0.74	1.15	8.37	4.91	4.92	2.74 ± 2.96
Bi	6.56	7.57	6.55	10.24	15.90	8.72	9.01	5.54	8.76 ± 3.27
Th	2.36	2.31	2.14	1.99	2.51	2.68	2.31	2.35	2.33 ± 0.21
U	1.59	1.77	1.77	1.59	1.50	1.68	0.99	1.45	1.54 ± 0.25

**Rare-earth elements**									

Y	0.98	0.97	0.94	1.18	1.16	3.16	0.85	1.08	1.29 ± 0.76
La	0.89	0.86	0.85	0.85	1.09	1.42	1.03	1.20	1.03 ± 0.21
Ce	1.00	0.98	0.97	0.89	1.21	2.94	1.41	2.93	1.54 ± 0.88
Pr	0.87	0.88	0.85	0.90	0.88	2.51	1.15	1.24	1.16 ± 0.57
Nd	0.80	0.79	0.77	0.82	0.69	1.01	0.60	0.55	0.75 ± 0.14
Sm	0.89	0.86	0.86	0.96	0.86	2.88	1.20	1.32	1.23 ± 0.69
Eu	0.53	0.60	0.53	0.65	0.61	1.06	0.68	0.73	0.67 ± 0.17
Gd	0.94	0.97	0.91	1.06	0.98	1.53	0.87	0.98	1.03 ± 0.21
Tb	0.99	0.97	0.92	1.10	0.80	1.90	0.98	1.25	1.11 ± 0.34
Dy	1.00	1.00	0.99	1.20	0.70	0.86	0.11	0.22	0.76 ± 0.41
Ho	0.96	1.05	0.93	1.14	0.90	1.77	0.90	1.14	1.10 ± 0.29
Er	1.11	1.15	1.05	1.32	1.19	3.10	1.00	1.34	1.41 ± 0.69
Tm	0.92	0.83	0.86	1.08	0.96	1.48	0.94	0.90	1.0±0.21
Yb	1.05	1.07	0.99	1.26	1.22	2.51	0.60	1.04	1.22 ± 0.56
Lu	0.95	0.85	0.90	1.12	0.99	1.96	0.77	1.06	1.07 ± 0.37

**Table 6:** The enrichment factor (EF_Fe_) of elements in surface SPM.

	Stations								
Element	1	2	3	4	5	6	7	8	Mean ± SD
**Major elements**									

Mg	0.36	0.46	0.47	0.52	0.86	10.88	25.13	43.75	10.3 ± 16.11
Al	2.16	2.15	2.15	1.83	1.33	0.95	1.91	2.09	1.82 ± 0.45
P	0.51	0.60	0.67	0.76	0.82	4.17	9.51	12.95	3.75 ± 4.86
K	0.74	0.80	0.86	0.88	0.97	5.57	12.19	19.42	5.18 ± 7.03
Ca	0.15	0.11	0.07	0.06	0.28	1.75	3.94	7.10	1.68 ± 2.58
Ti	0.50	0.48	0.40	0.30	0.26	0.46	0.78	0.98	0.52 ± 0.24
Mn	0.55	0.37	0.30	0.22	0.27	1.44	3.51	6.68	1.67 ± 2.31

**Trace elements**									

Li	8.35	8.72	8.64	7.39	4.60	7.08	14.29	23.87	10.37 ± 6.1
Sc	1.08	1.06	1.03	0.97	0.80	2.76	3.97	4.53	2.03 ± 1.51
V	0.91	0.92	1.03	1.08	1.18	3.24	6.30	3.15	2.23 ± 1.92
Cr	1.79	0.95	0.54	0.61	0.68	3.25	6.50	16.53	3.86 ± 5.51
Co	9.70	6.89	0.74	0.60	0.65	7.93	34.59	121.61	22.84 ± 41.44
Ni	6.41	4.79	0.86	0.90	0.95	7.04	20.51	53.76	11.9 ± 18.1
Cu	3.30	1.89	0.51	0.49	0.48	8.07	20.06	61.99	12.1 ± 21.23
Zn	0.69	0.70	0.70	0.54	0.48	0.95	1.72	4.03	1.23 ± 1.2
Ga	2.63	2.51	2.49	2.15	1.43	2.64	1.95	2.71	2.31 ± 0.44
As	1.45	1.33	1.17	1.20	1.48	4.91	2.33	57.77	8.96 ± 19.76
Rb	1.72	1.68	1.67	1.41	1.20	1.60	1.63	2.42	1.67 ± 0.35
Sr	0.30	0.30	0.30	0.36	0.99	5.98	13.79	25.20	5.9 ± 9.13
Zr	0.21	0.21	0.17	0.15	0.14	0.46	0.34	0.37	0.26 ± 0.12
Nb	0.84	0.78	0.45	0.36	0.34	1.26	1.58	2.02	0.95 ± 0.62
Mo	21.52	18.96	3.73	3.47	3.36	23.66	47.05	106.53	28.54 ± 34.78
Cd	0.23	0.17	0.12	0.03	0.06	0.46	0.00	0.00	0.13 ± 0.15
Sn	2.50	2.19	2.28	1.57	1.52	5.46	7.08	9.04	3.96 ± 2.87
Sb	0.55	0.54	0.51	0.47	0.48	1.75	1.28	2.18	0.97 ± 0.68
Cs	3.24	3.01	2.95	2.07	1.70	2.70	1.99	2.87	2.56 ± 0.57
Ba	0.68	0.61	0.48	0.52	0.57	1.27	0.70	7.08	1.49 ± 2.27
Hf	0.50	0.32	0.29	0.28	0.25	0.66	0.81	0.96	0.51 ± 0.27
W	7.51	4.99	3.44	2.99	2.91	9.66	10.53	12.52	6.82 ± 3.77
Tl	2.28	2.23	2.10	1.81	1.65	1.00	2.07	3.66	2.1 ± 0.75
Pb	1.33	1.31	1.26	1.35	1.54	7.92	9.36	10.29	4.29 ± 4.11
Bi	14.14	16.29	14.11	18.78	21.21	8.25	17.17	11.58	15.19 ± 4.1
Th	5.08	4.98	4.61	3.65	3.34	2.54	4.40	4.92	4.19 ± 0.92
U	3.42	3.81	3.81	2.91	2.00	1.59	1.89	3.03	2.81 ± 0.88

**Rare-earth elements**									

Y	2.12	2.08	2.03	2.17	1.55	2.99	1.61	2.26	2.1 ± 0.44
La	1.93	1.85	1.83	1.57	1.45	1.35	1.97	2.52	1.81 ± 0.37
Ce	2.15	2.11	2.09	1.64	1.62	2.78	2.69	6.11	2.65 ± 1.46
Pr	1.88	1.88	1.83	1.65	1.18	2.38	2.20	2.59	1.95 ± 0.44
Nd	1.71	1.70	1.65	1.50	0.93	0.96	1.15	1.14	1.34 ± 0.34
Sm	1.91	1.84	1.85	1.76	1.14	2.73	2.29	2.75	2.03 ± 0.54
Eu	1.14	1.29	1.14	1.19	0.82	1.01	1.29	1.52	1.17 ± 0.21
Gd	2.02	2.08	1.96	1.94	1.30	1.45	1.66	2.04	1.81 ± 0.3
Tb	2.12	2.09	1.98	2.02	1.07	1.80	1.88	2.61	1.94 ± 0.43
Dy	2.15	2.16	2.13	2.21	0.93	0.82	0.21	0.46	1.38 ± 0.86
Ho	2.07	2.26	2.01	2.10	1.20	1.67	1.72	2.39	1.93 ± 0.38
Er	2.40	2.48	2.27	2.42	1.58	2.93	1.90	2.81	2.35 ± 0.44
Tm	1.98	1.78	1.86	1.98	1.28	1.40	1.80	1.87	1.74 ± 0.26
Yb	2.27	2.30	2.13	2.32	1.62	2.37	1.14	2.17	2.04 ± 0.43
Lu	2.05	1.83	1.93	2.06	1.32	1.85	1.46	2.22	1.84 ± 0.31

**Table 7:** The enrichment factor (EF_Al_) of elements in surface sediments.

	Stations							
Element	2	3	4	5	6	7	8	Mean ± SD
**Major elements**								

P	0.54	0.46	0.40	0.44	0.43	0.59	0.66	0.5 ± 0.1
S	0.76	0.80	0.94	1.79	1.37	2.58	0.90	1.31 ± 0.67
K	0.39	0.37	0.73	0.48	0.39	0.66	0.68	0.53 ± 0.16
Ca	0.13	0.15	0.22	0.25	0.22	4.02	1.88	0.98 ± 1.48
Ti	0.56	0.50	0.54	0.60	0.53	0.78	0.81	0.62 ± 0.13
Mn	0.49	0.28	0.50	0.31	0.22	0.54	0.64	0.43 ± 0.15
Fe	0.63	0.60	0.52	0.62	0.55	0.81	0.83	0.65 ± 0.12

**Trace elements**								

Li	0.44	0.43	0.53	0.50	0.46	0.95	0.89	0.6 ± 0.22
Be	0.63	0.56	0.61	0.61	0.62	0.70	0.71	0.64 ± 0.05
Sc	0.77	0.71	0.63	0.79	0.70	0.88	0.91	0.77 ± 0.1
V	0.50	0.48	0.48	0.47	0.45	0.66	0.67	0.53 ± 0.09
Cr	0.33	0.33	0.31	0.31	0.29	0.62	0.70	0.41 ± 0.17
Co	0.32	0.26	0.26	0.32	0.28	0.49	0.60	0.36 ± 0.13
Ni	0.23	0.21	0.21	0.20	0.20	0.42	0.53	0.29 ± 0.13
Cu	0.32	0.29	0.30	0.26	0.28	0.28	0.38	0.3 ± 0.04
Zn	0.81	0.76	0.74	0.80	0.80	0.97	0.99	0.84 ± 0.1
Ga	1.01	0.97	0.92	0.98	0.97	0.97	0.94	0.97 ± 0.03
As	1.54	1.41	1.15	1.17	1.00	1.47	0.88	1.23 ± 0.25
Rb	0.68	0.63	1.07	0.77	0.69	1.06	1.04	0.85 ± 0.2
Sr	0.14	0.14	0.21	0.18	0.17	2.10	0.91	0.55 ± 0.74
Zr	0.36	0.33	0.39	0.44	0.35	0.55	0.44	0.41 ± 0.08
Nb	1.15	1.04	1.10	1.18	1.17	1.27	1.31	1.17 ± 0.09
Mo	1.27	0.88	0.81	0.72	0.95	0.23	0.18	0.72 ± 0.39
Ag	0.88	0.66	0.00	0.87	0.83	0.00	0.00	0.46 ± 0.44
Cd	0.00	0.31	0.00	0.28	0.33	0.00	0.00	0.13 ± 0.16
Sn	0.08	0.07	0.07	0.07	0.06	0.06	0.09	0.07 ± 0.01
Sb	0.57	0.56	0.57	0.61	0.49	0.71	0.68	0.6 ± 0.08
Cs	1.71	1.55	1.59	1.56	1.55	1.91	1.79	1.67 ± 0.14
Ba	0.31	0.28	0.48	0.31	0.29	0.51	0.52	0.39 ± 0.11
Hf	0.65	0.63	0.87	0.83	0.81	0.96	0.72	0.78 ± 0.12
W	0.75	0.65	0.73	0.71	0.71	0.71	0.62	0.7 ± 0.05
Hg	0.20	0.16	0.20	0.17	0.08	0.13	0.14	0.15 ± 0.04
Tl	0.49	0.45	0.64	0.52	0.51	0.61	0.55	0.54 ± 0.07
Pb	1.98	1.87	1.98	2.03	2.19	1.83	2.07	2.0 ± 0.12
Bi	34.33	22.53	21.49	3.08	3.40	1.96	2.05	12.69 ± 13.22
Th	2.38	2.21	2.01	2.22	2.29	2.05	1.71	2.12 ± 0.23
U	1.41	1.13	1.22	1.42	1.38	1.49	0.98	1.29 ± 0.18

**Rare-earth elements**								

Y	0.87	0.74	0.75	0.94	0.90	0.75	0.65	0.8 ± 0.1
La	1.06	0.92	1.20	1.17	1.12	1.60	1.50	1.23 ± 0.23
Ce	1.11	1.02	1.29	1.24	1.24	1.67	1.52	1.3 ± 0.23
Pr	0.97	0.85	1.11	1.10	1.04	1.44	1.33	1.12 ± 0.2
Nd	0.97	0.84	1.07	1.11	1.01	1.40	1.30	1.1 ± 0.19
Sm	1.07	0.92	1.05	1.23	1.13	1.38	1.28	1.15 ± 0.15
Eu	0.68	0.58	0.72	0.76	0.70	1.05	1.08	0.8 ± 0.19
Gd	1.04	0.88	0.94	1.16	1.06	1.16	1.04	1.04 ± 0.11
Tb	0.93	0.80	0.83	1.00	1.00	1.02	0.91	0.93 ± 0.09
Dy	1.13	0.97	1.00	1.21	1.17	1.09	0.96	1.08 ± 0.1
Ho	0.86	0.74	0.76	0.92	0.92	0.80	0.70	0.81 ± 0.09
Er	0.76	0.65	0.66	0.81	0.79	0.69	0.60	0.71 ± 0.08
Tm	0.74	0.64	0.68	0.78	0.77	0.68	0.62	0.7 ± 0.06
Yb	0.80	0.70	0.72	0.86	0.83	0.73	0.62	0.75 ± 0.08
Lu	0.73	0.64	0.66	0.81	0.78	0.64	0.58	0.69 ± 0.08

**Table 8:** The enrichment factor (EF_Fe_) of elements in surface sediments.

	Stations							
Element	2	3	4	5	6	7	8	Mean ± SD
**Major elements**								

Al	1.60	1.67	1.90	1.62	1.83	1.24	1.21	1.58 ± 0.27
P	0.86	0.76	0.76	0.72	0.79	0.72	0.80	0.77 ± 0.05
S	1.22	1.34	1.79	2.90	2.51	3.19	1.09	2.01 ± 0.86
K	0.62	0.61	1.39	0.77	0.71	0.82	0.83	0.82 ± 0.27
Ca	0.21	0.25	0.42	0.40	0.39	4.97	2.27	1.27 ± 1.79
Ti	0.89	0.84	1.03	0.98	0.96	0.97	0.98	0.95 ± 0.06
Mn	0.79	0.47	0.95	0.50	0.41	0.67	0.77	0.65 ± 0.2

**Trace elements**								

Li	0.71	0.72	1.01	0.82	0.84	1.17	1.08	0.91 ± 0.18
Be	1.01	0.94	1.17	1.00	1.13	0.87	0.86	1.0 ± 0.12
Sc	1.22	1.18	1.20	1.28	1.28	1.09	1.10	1.19 ± 0.08
V	0.80	0.80	0.91	0.76	0.82	0.82	0.81	0.82 ± 0.04
Cr	0.53	0.55	0.59	0.51	0.53	0.77	0.84	0.62 ± 0.13
Co	0.52	0.43	0.50	0.52	0.51	0.61	0.73	0.54 ± 0.1
Ni	0.37	0.36	0.40	0.33	0.36	0.52	0.64	0.42 ± 0.11
Cu	0.51	0.49	0.57	0.41	0.51	0.35	0.46	0.47 ± 0.07
Zn	1.29	1.26	1.41	1.29	1.45	1.20	1.20	1.3 ± 0.1
Ga	1.62	1.63	1.75	1.60	1.77	1.19	1.14	1.53 ± 0.26
As	2.46	2.36	2.18	1.90	1.82	1.82	1.07	1.94 ± 0.47
Rb	1.08	1.05	2.03	1.25	1.27	1.31	1.25	1.32 ± 0.33
Sr	0.23	0.24	0.39	0.30	0.31	2.60	1.10	0.74 ± 0.88
Zr	0.57	0.55	0.74	0.71	0.64	0.69	0.53	0.63 ± 0.08
Nb	1.83	1.73	2.10	1.91	2.14	1.57	1.58	1.84 ± 0.23
Mo	2.04	1.47	1.55	1.16	1.73	0.29	0.22	1.21 ± 0.7
Ag	1.40	1.10	0.00	1.41	1.51	0.00	0.00	0.77 ± 0.74
Cd	0.00	0.51	0.00	0.45	0.60	0.00	0.00	0.22 ± 0.28
Sn	0.13	0.11	0.13	0.11	0.12	0.08	0.10	0.11 ± 0.02
Sb	0.92	0.94	1.09	1.00	0.89	0.88	0.82	0.93 ± 0.09
Cs	2.73	2.60	3.03	2.53	2.83	2.36	2.16	2.61 ± 0.29
Ba	0.49	0.46	0.90	0.51	0.52	0.64	0.63	0.59 ± 0.15
Hf	1.05	1.06	1.66	1.34	1.48	1.18	0.87	1.23 ± 0.28
W	1.20	1.09	1.39	1.14	1.30	0.88	0.75	1.11 ± 0.23
Hg	0.31	0.27	0.37	0.27	0.15	0.16	0.17	0.24 ± 0.09
Tl	0.78	0.76	1.22	0.85	0.92	0.75	0.66	0.85 ± 0.18
Pb	3.17	3.14	3.78	3.29	4.01	2.26	2.51	3.16 ± 0.63
Bi	54.91	37.70	40.94	5.00	6.20	2.43	2.48	21.38 ± 22.31
Th	3.81	3.70	3.82	3.60	4.18	2.54	2.06	3.39 ± 0.78
U	2.26	1.88	2.32	2.30	2.52	1.84	1.19	2.04 ± 0.45

**Rare-earth elements**								

Y	1.39	1.23	1.44	1.52	1.65	0.93	0.79	1.28 ± 0.32
La	1.69	1.55	2.30	1.89	2.04	1.99	1.82	1.9 ± 0.24
Ce	1.77	1.71	2.46	2.00	2.27	2.07	1.83	2.02 ± 0.27
Pr	1.56	1.42	2.11	1.78	1.90	1.79	1.60	1.74 ± 0.23
Nd	1.54	1.41	2.03	1.81	1.85	1.73	1.57	1.71 ± 0.21
Sm	1.71	1.54	2.00	1.99	2.06	1.70	1.54	1.79 ± 0.22
Eu	1.09	0.97	1.37	1.24	1.28	1.30	1.30	1.22 ± 0.14
Gd	1.66	1.47	1.79	1.89	1.93	1.44	1.26	1.63 ± 0.25
Tb	1.49	1.35	1.58	1.62	1.83	1.27	1.10	1.46 ± 0.24
Dy	1.81	1.62	1.90	1.96	2.14	1.35	1.16	1.71 ± 0.35
Ho	1.37	1.25	1.45	1.50	1.67	0.99	0.85	1.3 ± 0.29
Er	1.21	1.09	1.26	1.31	1.45	0.85	0.73	1.13 ± 0.26
Tm	1.19	1.07	1.29	1.26	1.41	0.84	0.75	1.12 ± 0.24
Yb	1.28	1.18	1.37	1.39	1.52	0.91	0.75	1.2 ± 0.28
Lu	1.17	1.07	1.27	1.31	1.43	0.80	0.70	1.11 ± 0.27

Tables 9 and 10 show the distribution and mean values of the geoaccumulation index (I_geo_) of the chemical elements in surface SPM and surface bottom sediments along the salinity gradient.

**Table 9:** Geoaccumulation Index (I_geo_) of elements in surface SPM.

	Stations								
Element	1	2	3	4	5	6	7	8	Mean
**Major elements**									

Mg	−2,36	−2,01	−1,94	−1,65	−1,05	0,30	0,39	0,32	−1,00
Al	0,21	0,22	0,24	0,16	−0,41	−3,22	−3,33	−4,07	−1,28
P	−1,86	−1,61	−1,45	−1,10	−1,12	−1,08	−1,01	−1,44	−1,34
K	−1,34	−1,21	−1,08	−0,91	−0,87	−0,67	−0,65	−0,85	−0,95
Ca	−3,68	−4,06	−4,62	−4,66	−2,67	−2,34	−2,28	−2,30	−3,33
Ti	−1,91	−1,96	−2,18	−2,44	−2,75	−4,26	−4,61	−5,17	−3,16
Mn	−1,77	−2,34	−2,62	−2,88	−2,70	−2,62	−2,45	−2,39	−2,47
Fe	−0,90	−0,89	−0,87	−0,71	−0,83	−3,14	−4,26	−5,13	−2,09

**Trace elements**									

Li	2,16	2,24	2,24	2,17	1,37	−0,32	−0,42	−0,56	1,11
Sc	−0,78	−0,80	−0,82	−0,76	−1,14	−1,68	−2,27	−2,95	−1,40
V	−1,04	−1,01	−0,82	−0,60	−0,59	−1,45	−1,61	−3,48	−1,32
Cr	−0,06	−0,96	−1,77	−1,44	−1,38	−1,45	−1,56	−1,09	−1,21
Co	2,38	1,90	−1,30	−1,46	−1,46	−0,16	0,85	1,79	0,32
Ni	1,78	1,37	−1,09	−0,86	−0,91	−0,33	0,10	0,62	0,08
Cu	0,82	0,03	−1,85	−1,73	−1,88	−0,13	0,07	0,82	−0,48
Zn	−1,43	−1,40	−1,38	−1,59	−1,89	−3,22	−3,48	−3,12	−2,19
Ga	0,50	0,44	0,44	0,39	−0,31	−1,74	−3,29	−3,70	−0,91
As	−0,36	−0,48	−0,64	−0,45	−0,26	−0,85	−3,04	0,72	−0,67
Rb	−0,12	−0,14	−0,13	−0,22	−0,57	−2,47	−3,55	−3,86	−1,38
Sr	−2,63	−2,61	−2,59	−2,19	−0,84	−0,56	−0,47	−0,48	−1,55
Zr	−3,13	−3,17	−3,40	−3,41	−3,66	−4,28	−5,82	−6,57	−4,18
Nb	−1,15	−1,25	−2,03	−2,17	−2,38	−2,81	−3,60	−4,12	−2,44
Mo	3,53	3,36	1,03	1,08	0,92	1,42	1,30	1,60	1,78
Cd	−3,02	−3,47	−3,88	−5,99	−4,86	−4,28	−	−	−4,25
Sn	0,42	0,24	0,32	−0,06	−0,23	−0,70	−1,44	−1,96	−0,42
Sb	−1,76	−1,77	−1,83	−1,81	−1,88	−2,34	−3,91	−4,01	−2,41
Cs	0,80	0,70	0,69	0,33	−0,07	−1,71	−3,27	−3,61	−0,77
Ba	−1,45	−1,61	−1,93	−1,66	−1,65	−2,80	−4,78	−2,31	−2,27
Hf	−1,90	−2,53	−2,64	−2,57	−2,83	−3,75	−4,57	−5,19	−3,25
W	2,01	1,43	0,91	0,86	0,71	0,13	−0,86	−1,49	0,46
Tl	0,29	0,27	0,20	0,14	−0,10	−3,14	−3,21	−3,26	−1,10
Pb	−0,49	−0,50	−0,54	−0,28	−0,21	−0,16	−1,03	−1,77	−0,62
Bi	2,92	3,14	2,95	3,52	3,58	−0,10	−0,16	−1,60	1,78
Th	1,45	1,43	1,34	1,15	0,91	−1,80	−2,12	−2,84	−0,06
U	0,87	1,04	1,06	0,83	0,17	−2,48	−3,34	−3,53	−0,67

**Rare-earth elements**									

Y	0,18	0,17	0,15	0,40	−0,20	−1,57	−3,57	−3,96	−1,05
La	0,05	0,00	0,00	−0,07	−0,29	−2,72	−3,28	−3,80	−1,26
Ce	0,20	0,19	0,19	−0,01	−0,14	−1,67	−2,83	−2,52	−0,82
Pr	0,01	0,03	0,00	0,01	−0,60	−1,89	−3,12	−3,76	−1,17
Nd	−0,12	−0,12	−0,15	−0,13	−0,94	−3,21	−4,06	−4,95	−1,71
Sm	0,03	−0,01	0,02	0,10	−0,64	−1,70	−3,06	−3,67	−1,12
Eu	−0,71	−0,52	−0,68	−0,47	−1,12	−3,14	−3,89	−4,53	−1,88
Gd	0,12	0,17	0,10	0,24	−0,45	−2,61	−3,53	−4,10	−1,26
Tb	0,19	0,18	0,12	0,30	−0,73	−2,30	−3,35	−3,75	−1,17
Dy	0,20	0,22	0,22	0,43	−0,93	−3,43	−6,53	−6,26	−2,01
Ho	0,15	0,29	0,13	0,35	−0,57	−2,40	−3,48	−3,88	−1,17
Er	0,36	0,42	0,31	0,56	−0,17	−1,59	−3,33	−3,64	−0,88
Tm	0,09	−0,05	0,03	0,27	−0,48	−2,66	−3,41	−4,23	−1,31
Yb	0,28	0,31	0,22	0,50	−0,13	−1,90	−4,07	−4,01	−1,10
Lu	0,14	−0,01	0,08	0,33	−0,43	−2,25	−3,71	−3,98	−1,23

**Table 10:** Geoaccumulation Index (I_geo_) of elements in surface sediments.

	Stations							
Element	2	3	4	5	6	7	8	Mean
**Major elements**								

Al	0,03	0,10	−0,59	−0,02	0,12	−0,64	−0,50	−0,22
P	−0,87	−1,03	−1,93	−1,20	−1,10	−1,42	−1,10	−1,24
S	−0,36	−0,22	−0,68	0,82	0,58	0,72	−0,65	0,03
K	−1,34	−1,35	−1,05	−1,09	−1,24	−1,24	−1,05	−1,19
Ca	−2,93	−2,64	−2,77	−2,03	−2,09	1,36	0,41	−1,53
Ti	−0,82	−0,90	−1,48	−0,75	−0,81	−1,00	−0,79	−0,94
Mn	−1,00	−1,73	−1,61	−1,73	−2,04	−1,53	−1,14	−1,54
Fe	−0,65	−0,64	−1,52	−0,72	−0,75	−0,95	−0,77	−0,86

**Trace elements**								

Li	−1,14	−1,12	−1,51	−1,01	−0,99	−0,72	−0,66	−1,02
Be	−0,64	−0,73	−1,30	−0,72	−0,57	−1,16	−0,98	−0,87
Sc	−0,36	−0,41	−1,26	−0,37	−0,39	−0,82	−0,64	−0,61
V	−0,97	−0,96	−1,67	−1,12	−1,03	−1,25	−1,08	−1,15
Cr	−1,56	−1,50	−2,27	−1,71	−1,66	−1,32	−1,02	−1,58
Co	−1,60	−1,87	−2,53	−1,67	−1,72	−1,67	−1,23	−1,75
Ni	−2,10	−2,12	−2,84	−2,33	−2,23	−1,91	−1,42	−2,14
Cu	−1,63	−1,68	−2,33	−1,99	−1,73	−2,48	−1,89	−1,96
Zn	−0,28	−0,31	−1,03	−0,35	−0,21	−0,69	−0,51	−0,48
Ga	0,05	0,06	−0,71	−0,04	0,07	−0,70	−0,58	−0,27
As	0,65	0,59	−0,40	0,20	0,12	−0,09	−0,68	0,06
Rb	−0,54	−0,57	−0,50	−0,40	−0,41	−0,56	−0,44	−0,49
Sr	−2,76	−2,70	−2,88	−2,47	−2,45	0,43	−0,64	−1,93
Zr	−1,45	−1,51	−1,96	−1,21	−1,39	−1,49	−1,69	−1,53
Nb	0,23	0,15	−0,45	0,21	0,35	−0,30	−0,11	0,01
Mo	0,38	−0,09	−0,89	−0,51	0,05	−2,76	−2,94	−0,97
Ag	−0,16	−0,50	−	−0,23	−0,15	−	−	−0,26
Cd	−	−1,61	−	−1,87	−1,48	−	−	−1,65
Sn	−3,56	−3,79	−4,44	−3,91	−3,85	−4,59	−4,05	−4,03
Sb	−0,77	−0,73	−1,40	−0,73	−0,92	−1,13	−1,06	−0,96
Cs	0,80	0,73	0,08	0,62	0,75	0,29	0,34	0,52
Ba	−1,68	−1,76	−1,67	−1,69	−1,69	−1,60	−1,43	−1,65
Hf	−0,58	−0,56	−0,79	−0,30	−0,18	−0,71	−0,98	−0,59
W	−0,38	−0,51	−1,05	−0,53	−0,37	−1,14	−1,19	−0,74
Hg	−2,33	−2,54	−2,94	−2,62	−3,46	−3,64	−3,30	−2,97
Tl	−1,01	−1,04	−1,24	−0,96	−0,86	−1,37	−1,36	−1,12
Pb	1,02	1,00	0,39	1,00	1,25	0,23	0,56	0,78
Bi	5,13	4,59	3,83	1,60	1,88	0,33	0,54	2,56
Th	1,28	1,24	0,41	1,13	1,32	0,39	0,28	0,86
U	0,53	0,27	−0,31	0,48	0,58	−0,07	−0,52	0,14

**Rare-earth elements**								

Y	−0,17	−0,34	−1,00	−0,11	−0,03	−1,05	−1,11	−0,55
La	0,11	−0,02	−0,33	0,20	0,28	0,04	0,09	0,05
Ce	0,18	0,13	−0,23	0,28	0,43	0,10	0,11	0,14
Pr	−0,01	−0,14	−0,45	0,12	0,18	−0,12	−0,09	−0,07
Nd	−0,02	−0,15	−0,50	0,13	0,14	−0,16	−0,12	−0,10
Sm	0,13	−0,02	−0,52	0,27	0,29	−0,18	−0,14	−0,03
Eu	−0,53	−0,69	−1,07	−0,41	−0,39	−0,57	−0,39	−0,58
Gd	0,08	−0,09	−0,68	0,20	0,20	−0,43	−0,44	−0,17
Tb	−0,07	−0,22	−0,87	−0,03	0,12	−0,61	−0,64	−0,33
Dy	0,21	0,05	−0,60	0,25	0,35	−0,52	−0,56	−0,12
Ho	−0,20	−0,33	−0,99	−0,14	−0,01	−0,96	−1,01	−0,52
Er	−0,38	−0,52	−1,19	−0,33	−0,21	−1,19	−1,23	−0,72
Tm	−0,40	−0,55	−1,16	−0,38	−0,25	−1,21	−1,18	−0,73
Yb	−0,30	−0,41	−1,07	−0,25	−0,15	−1,09	−1,19	−0,64
Lu	−0,42	−0,54	−1,18	−0,33	−0,23	−1,28	−1,28	−0,75

Tables 11 and 12 show the partitioning coefficient *K*_SPM/Water_ (or *K*d) calculated as the ratio of particulate-to-filtered element concentrations and lg *K*d and illustrate the distribution of elements between the dissolved and particulate phases in the surface water layer.

**Table 11:** The partitioning coefficient (*K*_SPM/Water_).

	Stations (Salinity, ‰)				
Element	1 (< 0.01)	3 (8.49)	4 (15.82)	7 (33.12)	8 (33.56)
Li	56565	6158	2000	57	55
Na	92	11	8	23	22
Mg	2551	64	25	16	16
K	5612	520	191	34	31
Ca	865	47	17	17	18
Rb	16662	8036	3764	82	71
Sr	1716	103	49	28	30
Mo	261645	9894	4391	982	1245
Cs	169390	80156	59998	3313	2861
Ba	13628	7401	10664	4277	22607
U	86535	50063	16877	124	116

**Table 12:** The partitioning coefficient (lg (*K*_SPM/Water_)).

	Stations (Salinity, ‰)				
Element	1 (< 0.01)	3 (8.49)	4 (15.82)	7 (33.12)	8 (33.56)
Li	4.75	3.79	3.30	1.75	1.74
Na	1.96	1.05	0.91	1.37	1.34
Mg	3.41	1.81	1.39	1.21	1.21
K	3.75	2.72	2.28	1.53	1.49
Ca	2.94	1.67	1.23	1.23	1.26
Rb	4.22	3.91	3.58	1.91	1.85
Sr	3.23	2.01	1.69	1.44	1.47
Mo	5.42	4.00	3.64	2.99	3.10
Cs	5.23	4.90	4.78	3.52	3.46
Ba	4.13	3.87	4.03	3.63	4.35
U	4.94	4.70	4.23	2.09	2.07

Table 13 reports on the partitioning coefficient *K*_SPM/Sed_ calculated as the ratio of the element content in surface SPM and surface sediment and illustrates the distribution of elements between the particulate and sedimentary phases.

**Table 13:** The partitioning coefficient (*K*_SPM/Sed_).

	Stations							
Element	2	3	4	5	6	7	8	Mean ± SD
**Major elements**								

Al	1.24	1.20	1.84	0.83	0.11	0.17	0.09	0.78 ± 0.68
P	1.72	2.15	5.07	3.04	2.89	3.80	2.27	2.99 ± 1.14
K	0.70	0.76	0.70	0.74	0.95	0.95	0.73	0.79 ± 0.11
Ca	0.76	0.43	0.45	1.07	1.42	0.13	0.26	0.65 ± 0.46
Ti	0.43	0.39	0.49	0.24	0.09	0.08	0.05	0.25 ± 0.19
Mn	0.78	1.07	0.82	1.01	1.32	1.05	0.83	0.98 ± 0.19
Fe	1.04	1.05	2.16	1.14	0.23	0.12	0.06	0.83 ± 0.75

**Trace elements**								

Li	1.34	1.33	1.65	0.67	0.21	0.16	0.14	0.78 ± 0.65
Be	1.26	1.30	1.64	1.05	0.50	0.09	0.06	0.84 ± 0.63
Sc	1.03	1.05	1.98	0.82	0.57	0.51	0.28	0.89 ± 0.56
V	0.97	1.09	2.08	1.44	0.75	0.77	0.19	1.04 ± 0.6
Cr	2.19	1.20	2.58	1.81	1.67	1.23	1.38	1.72 ± 0.52
Co	13.40	1.75	2.48	1.37	3.49	6.78	9.61	5.55 ± 4.57
Ni	12.16	2.24	4.33	2.94	4.08	4.40	4.50	4.95 ± 3.29
Cu	5.34	1.50	2.55	1.83	5.09	9.86	11.05	5.32 ± 3.83
Zn	1.01	1.04	1.48	0.76	0.27	0.32	0.36	0.75 ± 0.46
Ga	1.25	1.24	2.05	0.79	0.27	0.16	0.11	0.84 ± 0.72
As	1.28	1.19	2.69	2.02	1.43	0.36	7.35	2.33 ± 2.33
Rb	0.74	0.76	0.68	0.50	0.13	0.07	0.05	0.42 ± 0.32
Sr	0.69	0.67	1.00	1.93	2.31	0.33	0.70	1.09 ± 0.74
Zr	0.30	0.27	0.37	0.18	0.13	0.05	0.03	0.19 ± 0.13
Nb	0.44	0.27	0.37	0.20	0.14	0.13	0.08	0.23 ± 0.14
Mo	9.06	2.48	4.49	3.08	2.97	19.09	26.72	9.7 ± 9.54
Ag	2.45	2.38	−	2.34	2.01	−	−	−
Cd	−	1.07	−	0.65	0.74	−	−	−
Sn	1.07	1.32	1.59	0.98	0.68	0.68	0.32	0.95 ± 0.43
Sb	0.73	0.68	1.10	0.65	0.55	0.21	0.19	0.59 ± 0.32
Cs	1.17	1.21	1.49	0.78	0.23	0.11	0.08	0.72 ± 0.59
Ba	0.94	0.80	0.91	0.93	0.42	0.10	0.49	0.65 ± 0.32
Hf	0.37	0.34	0.42	0.25	0.12	0.10	0.08	0.24 ± 0.14
W	3.88	2.98	4.18	2.61	1.56	1.34	0.90	2.49 ± 1.27
Tl	0.92	0.90	0.99	0.69	0.08	0.11	0.10	0.54 ± 0.43
Pb	1.07	1.05	1.91	1.33	1.15	1.27	0.61	1.2 ± 0.39
Bi	0.50	0.63	1.59	7.77	0.50	1.41	0.45	1.83 ± 2.66
Th	1.12	1.08	1.69	0.87	0.12	0.18	0.12	0.74 ± 0.61
U	1.28	1.54	1.96	0.72	0.11	0.09	0.11	0.83 ± 0.77

**Rare-earth elements**								

Y	1.07	1.19	2.23	0.79	0.29	0.15	0.12	0.83 ± 0.75
La	1.08	1.18	1.40	0.83	0.15	0.12	0.08	0.69 ± 0.56
Ce	1.06	1.10	1.23	0.79	0.24	0.14	0.17	0.67 ± 0.48
Pr	1.03	1.11	1.38	0.61	0.24	0.13	0.08	0.65 ± 0.53
Nd	0.97	1.04	1.34	0.49	0.10	0.07	0.04	0.58 ± 0.54
Sm	0.98	1.10	1.65	0.57	0.27	0.15	0.09	0.69 ± 0.58
Eu	1.08	1.09	1.63	0.66	0.16	0.11	0.06	0.68 ± 0.61
Gd	1.08	1.15	1.91	0.65	0.14	0.12	0.08	0.73 ± 0.69
Tb	1.15	1.22	2.16	0.59	0.18	0.14	0.11	0.79 ± 0.76
Dy	1.07	1.20	2.17	0.47	0.08	0.02	0.02	0.72 ± 0.81
Ho	1.18	1.17	2.15	0.63	0.16	0.15	0.12	0.79 ± 0.76
Er	1.14	1.17	2.21	0.73	0.25	0.15	0.12	0.83 ± 0.75
Tm	0.97	1.13	2.04	0.71	0.14	0.16	0.09	0.75 ± 0.71
Yb	1.04	1.05	2.01	0.74	0.20	0.09	0.10	0.75 ± 0.7
Lu	0.97	1.12	2.08	0.68	0.18	0.14	0.11	0.75 ± 0.71

Table S1 of Supplementary materials (available from Mendeley Data) covers the source/raw, analysed, calculated data, and descriptive statistics on the bulk, normalized-to-Al, and normalized-to-Fe contents of 53 chemical elements along with environmental indices (EF_Al_, EF_Fe_, and I_geo_) and partition coefficient (*K*_SPM/Sed_, calculated of both bulk and normalized-to-Al element contents) in the surface SPM and surface bottom sediments of the Cai River estuary and includes the respective reference material values.

The principal component analysis (PCA) plot in Fig. 2a shows the spatial distribution of filtered elements along the salinity gradient. The PCA plot in Fig. 2**b** shows the spatial distribution of the partitioning coefficient *K*_SPM/Water_ (*K*d) along the salinity gradient.

**Fig. 2a:**
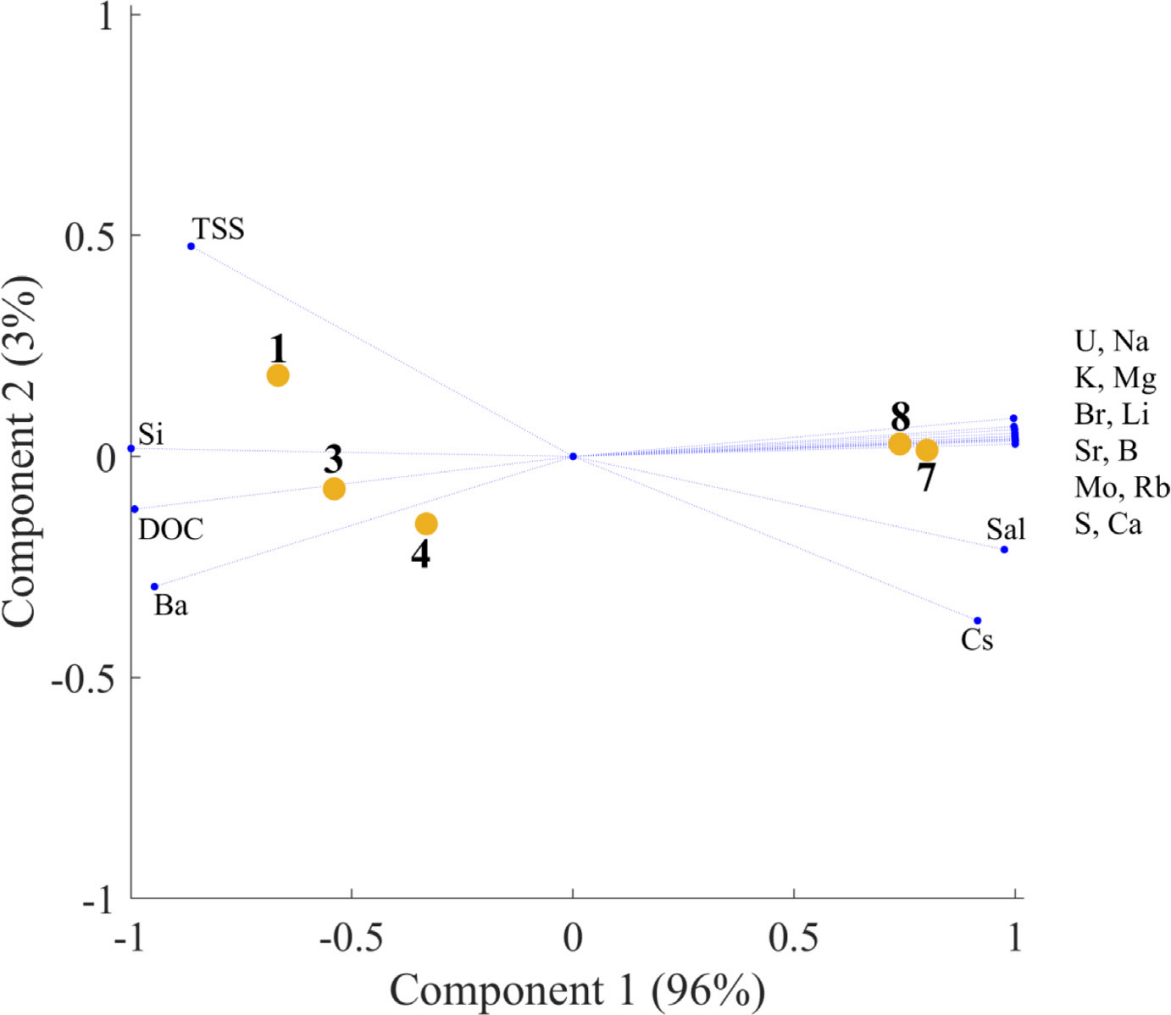
PCA plot for filtered elements.

**Fig. 2b:**
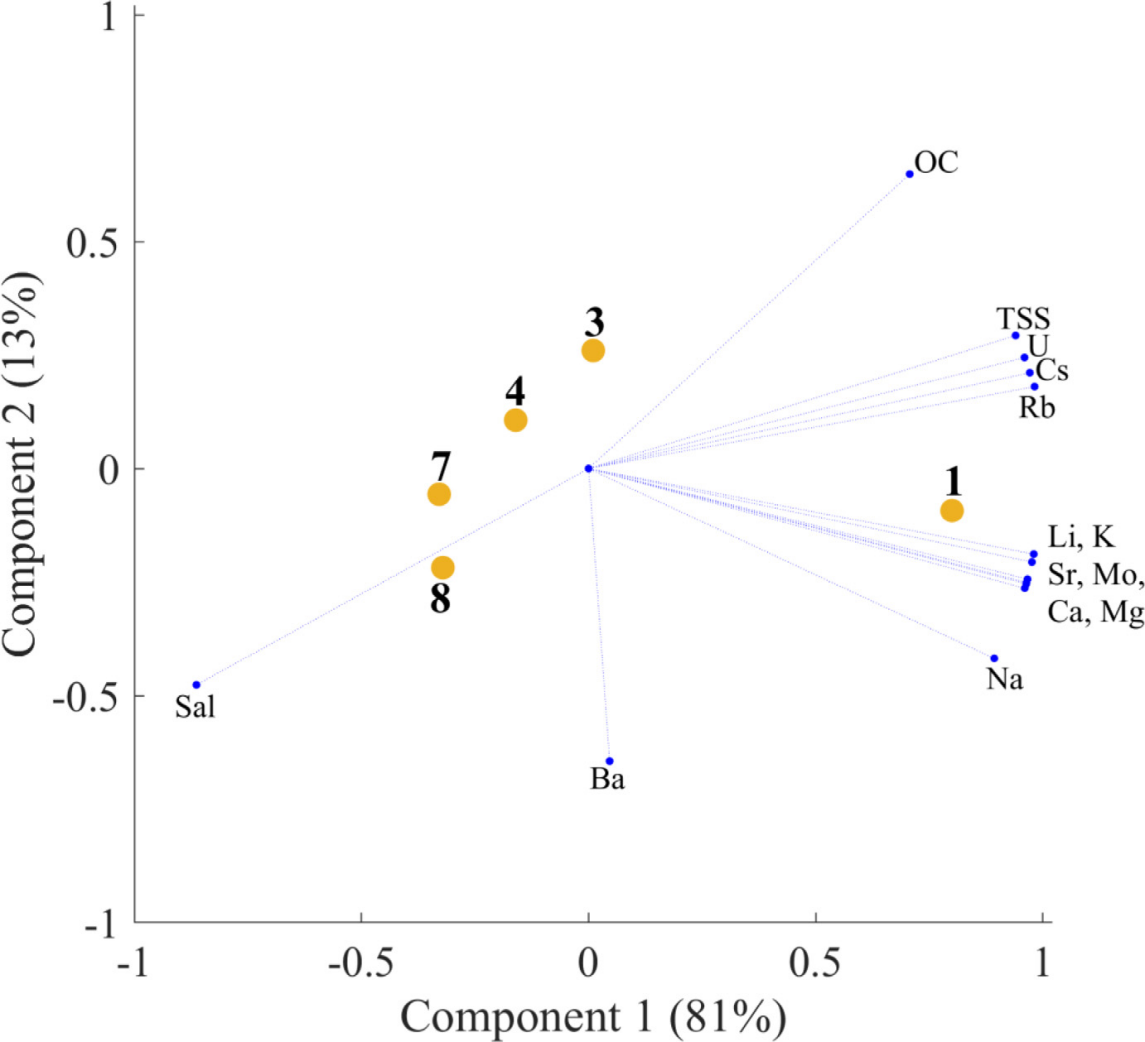
PCA plot for partitioning coefficient (*K*_SPM/Water_).

## 2 Experimental Design, Materials and Methods

### 2.1 Sampling

The surface water (eight locations, sts. 1–8) and surface sediment samples (seven locations, sts. 2–8) were collected in the Cai River estuary and Nha Trang Bay in July-August 2013 (Fig. 1). The surface water samples were collected using a plastic Niskin Bottle. The salinity and alkalinity were measured on board by portable conductivity apparatuses HI 98129 Combo and HI 98302 DIST 2 (Hanna Instruments, Germany). The suspended particulate matter (SPM) samples were obtained by filtering water samples in an all-glass filtering system. Pre-weighted polycarbonate filters (pore diameter 0.45 µm; Millipore) were used for total suspended sediment (TSS), combusted glass fiber filters (GF/F, Whatman) were used for POC, and acid-clean cellulose filters (pore diameter 0.45 µm; Millipore) were used for chemical composition analyses. After sampling, the polycarbonate and cellulose filters were rinsed with 250 mL of Milli-Q water to remove seawater salts. All filters were dried to constant weight at 60 °C. After filtration, the filtered water samples were placed in sterile polypropylene containers, acidified to pH 2 with 65% nitric acid (Merck) for dissolved (filtered) chemical elements (DCE), and with 35% hydrochloric acid (Merck) for DOC and TDN analyses and were kept cool until laboratory analyses were performed [1].

The surface sediment samples were collected by scuba divers with a manual plastic piston corer, which was produced at the Shirshov Institute of Oceanology, Russian Academy of Sciences (IO RAS). The samples were placed into pre-cleaned polyethylene containers using stainless steel spatulas. One portion of the sample was kept frozen until grain size and mineralogy analyses were performed. Another portion was dried to constant weight at 60 °C until chemical element analysis was performed.

All sampling, sampling transportation, and preparation procedures were performed using standard clean techniques according to the manual of [2].

### 2.2 Laboratory analyses

The organic geochemistry analyses of the filtered water, SPM and sediment samples were performed at the Ocean Chemistry Laboratory of the IO RAS. The DOC, POC, TDN, TC and TIC were determined with the analyser TOC 5000-V-CPH (Shimudzu Co., Japan). The DOC in the water samples was determined by high-temperature (680 °С) thermocatalytic oxidation with dispersion-free IP detection. The ТDN was determined by catalytic thermal decomposition followed by chemiluminescent detection [3]. C/N ratios were calculated by dividing DOC by TDN [1]. The TC contents in the SPM and sediment samples were determined by high-temperature (900°C) combustion in airflow using an SSM 5000 A device. The TIC contents were determined by dry burning at 200° С with H_3_PO_4_. The TOC contents were determined as the difference between TC and TIC contents in the samples. The error of element measurements accounted for 1 rel.%. The reproducibility of the data was within ±5% [3, 4]. Elemental analysis of the filtered water, SPM and sediment samples was performed at the Analytical and Certification Center of the Institute of Microelectronics Technology Problems and High Purity Materials, Russian Academy of Sciences. Inductively coupled plasma atomic emission spectrometry (ICP-AES) (ICAP-61, Thermo Jarrell Ash, USA) and inductively coupled plasma mass spectrometry (ICP-MS) (X-7, Thermo Elemental, USA) were used for the elemental analysis of the filtered water samples and the solution obtained by the total dissolution of SPM and sediment samples in HNO_3_ + HClO_4_ (3:1 by volume, Merck) in an autoclave system (Аnkon-АТ-2, Russia). A 50-mg weighed portion was placed in a Teflon reaction chamber, and 0.05 mL of a solution of a mixture of isotopic labels containing 8 mg L^−1 146^Nd, 5 mg L^−1 161^Dy and 3 mg L^−1 174^Yb was added. This solution was used to control the sample digestion by the "added-found" method. Then, 2 mL of HF (Hydrofluoric acid 40% GR, ISO, Merck) and 0.5 mL of HNO_3_ (Nitric acid 65%, max. 0.0000005% Hg, GR, ISO, Merck) were added, and the mixture was covered with a lid and left at room temperature for 6 h. Subsequently, the chambers were placed on a hotplate, heated up to 170–180 °C, and the solution was evaporated to dryness. After cooling, 2 mL of HF, 0.5 mL of HClO_4_ (Perchloric acid fuming 70% Supratur, Merck) and 0.2 mL of HNO_3_ were added to each chamber. The reaction chambers were sealed and fixed in the titanium body of the autoclave, and stepwise heating was carried out according to the following scheme: 160°C (60 min), 180 °C (60 min) and 200 °C (60 min). The pressure inside the reaction chamber was ∼16 MPa. After cooling, 1 mL of HNO_3_ and 1 mL of HCl (Hydrochloric acid faming 37% GR, ISP, Merck) were added to each sample. The reaction chambers were sealed and kept at a temperature of 160 °C for 60 min [5]. The Hg content was determined in separately prepared samples. For this, a 50 mg portion was treated for 30 min at 96 °C with a mixture of HCl + HNO_3_ (3:1 by volume) in an open system [5]. After cooling, all resulting solutions were transferred to polyethylene Eppendorf cups (Labcon, USA and Deltalab, Spain) and 0.2 mL of 10 mg L^−1^ In solution was added, which was used as an internal standard in mass spectral measurements. Then, the sample was brought to a volume of 10 mL using Milli-Q water. The solutions obtained by carrying out the above procedures without a sample portion were used as controls. Deionised water with a resistivity of 18.2 MΩ (Milli-Q) was used. Calibration curves were plotted using multi-element and single-element standard solutions (High-Purity Standards, USA). A detailed description of the autoclave digestion procedures and the analytical procedures for elemental analysis are given by [6,7].

The ICP-AES method was applied to determine major (Na, Mg, P, S, K and Ca) and some trace elements (Li, B, Al, Ti, V, Cr, Mn, Co, Fe, Ni, Cu, Zn, Sr and Ba). The ICP-MS method was used to determine only trace (Li, Be, B, Sc, V, Cr, Mn, Co, Ni, Cu, Zn, Ga, As, Se, Rb, Sr, Mo, Ag, Cd, Sn, Sb, Cs, Ba, Re, Au, Hg, Tl, Pb, Bi, Th and U) and rare earth elements (Y, La, Ce, Pr, Nd, Sm, Eu, Gd, Tb, Dy, Ho, Er, Tm, Yb and Lu). The simultaneous use of two independent analysis methods improves the quality and accuracy of the results obtained. First, the list of the analysed elements expands significantly. Second, an additional inter-method control of the measurement accuracy is performed for each sample when certain elements (Li, B, V, Cr, Mn, Co, Ni, Cu, Zn, Sr and Ba), whose contents in the sample are reliably determined by both methods (ICP-AES and ICP-MS), serve as internal standards to check method accuracy [7]. The combined use of ICP-AES and ICP-MS allows measurements to be validated by comparing the measurements of six elements by both methods simultaneously [6,7]. The error of the element measurements was no greater than 10–15 rel.% for ICP-AES and 10–30 rel.% for ICP-MS, depending on element content [7].

### 2.3 Accuracy of the analytical determinations

The precision and validity of the analytical determinations were evaluated using Certified Reference Materials (CRM) which were randomly allocated within the determinations. The CRM used included Certified Reference Material “Trace Metals in Drinking Water” (EU) for filtered water samples, Andesite, AGV-2 (United States Geological Survey) and Essexite Gabbro SRM-2A (Russian Geochemical Standard) for SPM and sediment samples. The discrepancy between the certified and measured element contents was within the limits of confidence intervals in every case. The limits of detection were calculated following [6] for all elements (Tables 2 and 3).

### 2.4 Calculation of enrichment factor and partition coefficients

The Enrichment Factor (EF) normalizes metal contents according to sediment texture properties [1,8]. In the present article, we used both Al and Fe as M_REF_ for the calculation of EF_Al_ and EF_Fe_, respectively. The EF was calculated as follows:

$$\text{EF}=\left(\left[M\right]/{\left[M_{\text{REF}}\right]}_{s}\right)/\left(\left[M\right]/{\left[M_{\text{REF}}\right]}_{b}\right),$$

where the [M]/[M_REF_]_s_ is the ratio of the concentrations of the metal to reference metal in the sample, and [M]/[M_REF_]_b_ is the ratio of the concentrations of the reference material. Average chemical composition of Suspended Particulate Matter in World Rivers (WRSPM) and average chemical composition of World Shale values were used as a background for SPM and sediments respectively [9,10].

EF values lower than 1.5 suggest that the element is derived mainly from natural sources, whereas EF values higher than 1.5 suggest anthropogenic sources [1].

The Geoaccumulation Index (I_geo_) was used to measure metal pollution in sediments and was calculated using the following equation [3,8]:

$$I_{\text{geo}}=\log_{2}\left(\text{Sample}/1.5\times \text{Background}\right),$$

where average WRSPM and shale values were used as a background for SPM and sediments, respectively [9,10]. I_geo_<0: uncontaminated; I_geo_<1: uncontaminated to moderately contaminated; I_geo_<2: moderately contaminated; I_geo_<3: moderately to highly contaminated, etc. [1,8].

The partitioning coefficient (*K*_SPM/Water_) was calculated as the ratio of element content in surface SPM and filtered water [1]. The partitioning coefficient (*K*_SPM/Sed_) was calculated as the ratio of element content in surface SPM and sediments [11,12].

### 2.5 Statistical analysis

Descriptive statistics were calculated using Microsoft Excel 2010. Principal component analysis was conducted using the MATLAB R2018a computing environment (MathWorks, Inc., USA). In all cases, PCA analyses were carried out on normalized data, i.e. for every variable its mean value was subtracted from the raw data and after that the obtained centered variables were normalized by their standard deviations.

## Ethics Statement

The authors declare that they have followed the general ethics rules of scientific research performance and publishing. All applicable international, national, and/or institutional guidelines for the care and use of animals were followed.

## CRediT authorship contribution statement

**Sofia E. Koukina:** Conceptualization, Investigation, Methodology, Writing – original draft. **Nikolay V. Lobus:** Investigation, Resources, Validation, Writing – review & editing. **Alexander V. Shatravin:** Software, Data curation, Formal analysis, Visualization.

## Conflict of Interest

The authors declare that they have no known competing financial interests or personal relationships that could have appeared to influence the work reported in this paper.

## Appendix Supplementary materials

Image, application 1
