# Supplementary material for: Dataset on the abundance, enrichment and partitioning of chemical elements between the filtered, particulate and sedimentary phases in the Cai River estuary (South China Sea)
Source: Data Brief. 2021 Sep 23;38:107412. doi: 10.1016/j.dib.2021.107412 (PMC8481967; doi:10.1016/j.dib.2021.107412)
Supplement: Supplementary file 1 [file mmc1.pdf]

Data in Brief

Dataset on the abundance, enrichment and partitioning of chemical elements between the filtered, particulate and sedimentary phases in the Cai River estuary (South China Sea)

--Manuscript Draft--

**Manuscript Number:**	DIB-D-21-01003
**Article Type:**	Data Article
**Keywords:**	South China Sea; Nha Trang Bay; organic geochemistry; major elements; trace elements; rare earth elements; enrichment factor; geoaccumulation index; partitioning coefficient
**Corresponding Author:**	Sofia Koukina, Dr.<br>FSBIS P P Shirshov Institute of Oceanology of the Russian Academy of Sciences: FGBUN Institut okeanologii imeni P P Sirsova Rossijskoj akademii nauk<br>RUSSIAN FEDERATION
**First Author:**	Sofia Koukina, Dr.
**Order of Authors:**	Sofia Koukina, Dr.
	Nikolay Lobus, Dr.
	Alexander Shatravin
**Abstract:**	This data article refers to the paper entitled “Multi-element signatures in solid and solution phases in a tropical mixing zone: A case study in the Cai River estuary, Vietnam” (Koukina et al., 2021), which considers the fate of major, trace, and rare-earth elements transported through the estuarine geochemical filter of the typical tropical estuary. The present work contributes to the local geochemical baselines as a background for long-term monitoring of potential hazardous elements. Therefore, the dataset covers the abundance, enrichment, and partitioning parameters of 54 chemical elements in the water, suspended particulate matter, and bottom sediment samples collected in the Cai River estuary and the adjacent part of the Nha Trang Bay (South China Sea) between July and August 2013. The total filtered, particulate, and sedimentary elements were determined by atomic emission and inductively coupled plasma mass spectrometry (ICP-AES; ICP-MS). The environmental indices (the enrichment factor and geo-accumulation index) and partition coefficients were calculated from the total element contents. The data provided is essential for the comprehensive environmental assessment of the anthropogenic impact on the coastal ecosystem as well as for the evaluation and modelling of element fractionation and mobility at the estuarine gradients.
**Suggested Reviewers:**	Pratima M. Kessarkar<br>pratimak@nio.org
	Sandra Costa-Böddeker<br>s.boeddeker@tu-bs.de
	Nguyen Thanh-Nho<br>ntnho@hcmus.edu.vn

Powered by Editorial Manager® and ProduXion Manager® from Aries Systems Corporation

Manuscript Click here to view linked References

Dataset on the abundance, enrichment and partitioning of chemical elements between the filtered, particulate and sedimentary phases in the Cai River estuary (South China Sea)

Sofia E. Koukina [1] (corresponding author) (ORCID 0000-0002-5969-1446),
Nikolay V. Lobus [2] (ORCID 0000-0002-2517-4061)
Alexander V. Shatravin [1, 3]

[1] Shirshov Institute of Oceanology, Russian Academy of Sciences
Nahimovskiy pr. 36, 117997 Moscow, Russia
[2] Timiryazev Institute of Plant Physiology, Russian Academy of Sciences, Botanicheskaya st. 35, 127276 Moscow, Russia
[3] Prokhorov General Physics Institute of the Russian Academy of Sciences
Vavilova st. 38, 119991 Moscow, Russia

Corresponding author(s)
Sofia E. Koukina (skoukina@gmail.com)

Abstract

This data article refers to the paper entitled "Multi-element signatures in solid and solution phases in a tropical mixing zone: A case study in the Cai River estuary, Vietnam" (Koukina et al., 2021), which considers the fate of major, trace, and rare-earth elements transported through the estuarine geochemical filter of the typical tropical estuary. The present work contributes to the local geochemical baselines as a background for long-term monitoring of potential hazardous elements. Therefore, the dataset covers the abundance, enrichment, and partitioning parameters of 54 chemical elements in the water, suspended particulate matter, and bottom sediment samples collected in the Cai River estuary and the adjacent part of the Nha Trang Bay (South China Sea) between July and August 2013. The total filtered, particulate, and sedimentary elements were determined by atomic emission and inductively coupled plasma mass spectrometry (ICP-AES; ICP-MS). The environmental indices (the enrichment factor and geo-accumulation index) and partition coefficients were calculated from the total element contents. The data provided is essential for the comprehensive environmental assessment of the anthropogenic impact on the coastal ecosystem as well as for the evaluation and modelling of element fractionation and mobility at the estuarine gradients.

Keywords: South China Sea, Nha Trang Bay, organic geochemistry, major elements, trace elements, rare earth elements, enrichment factor, geoaccumulation index, partitioning coefficient

Specifications Table

**Subject**	Environmental Chemistry
**Specific subject area**	The Environmental Geochemistry of Estuaries, Aquatic Geochemistry, Geochemical cycles of elements, Ocean Ecology
**Type of data**	Tables, Graphs, Figures
**How data were acquired**	TOC 5000-V-CPH analyzer (Shimudzu Co., Japan), ICP-AES (ICAP-61, Thermo Jarrell Ash, USA), ICP-MS (X-7, Thermo Elemental, USA), total dissolution in HNO_3 + $HClO_4$ (3:1 by volume, Merck) in an autoclave system (Ankon-AT-2, Russia),<br>Microsoft Excel 2010, MATLAB R2018a (MathWorks, Inc., USA)
**Data format**	Raw, Analyzed
**Parameters for data collection**	The surface water (eight locations, sts. 1–8) and surface sediment samples (seven locations, sts. 2–8) were collected in the Cai River estuary and Nha Trang Bay during the dry season in July-August 2013. The surface water samples were collected using a plastic Niskin Bottle. The suspended particulate matter (SPM) samples were obtained by filtering water samples in an all-glass filtering system. The surface sediment samples were collected by scuba divers with a manual plastic piston corer. All sampling, sampling transportation, and preparation procedures were performed using standard clean techniques.
**Description of data collection**	Organic geochemistry parameters in the filtered water, SPM and sediment samples (DOC, POC, TDN, TC and TIC) were determined with the analyser TOC 5000-V-CPH (Shimudzu Co., Japan). Standard methods (ICP-AES and ICP-MS) were applied to determine the content of the chemical elements. The elemental analysis of the filtered water samples and the solution obtained by the total dissolution of SPM and sediment samples was determined with the ICAP -61 (Thermo Jarrell Ash, USA) with the X-7 (Thermo Elemental, USA).
**Data source location**	The latitude and longitude of the sampling sites are given in Table 1. Preparation of the samples and data analysis were conducted at the

	P.P. Shirshov Institute of Oceanology, Russian Academy of Sciences, Moscow, Russia. The analytical procedure was carried out at the Institute of Microelectronics Technology and High Purity Materials, Russian Academy of Sciences, Chernogolovka, Moscow Region, Russia.
**Data accessibility**	With the article
**Related research article**	Koukina S.E., Lobus N.V., Shatravin A.V. Multi-element signatures in solid and solution phases in a tropical mixing zone: A case study in the Cai River estuary, Vietnam // Chemosphere. 2021. V. 280. 130951. https://doi.org/10.1016/j.chemosphere.2021.130951

Value of the Data

- The data on element enrichment and partitioning in the solid and solution phases of the typical tropical estuary is essential for an adequate assessment of hazardous elements, their possible pathways, and the potential risk to the coastal environment under multiple pressures.
- The data is useful for environmental scientists as well as for decision makers in order to prevent chemical pollution and implement sustainable development goals in the coastal Vietnam.
- The data may be used for the evaluation of the local geochemical background and further environmental monitoring and assessment of the developing Nha Trang Bay region as well as for the global modelling of element fractionation and mobility at the estuarine gradients.

Data Description

Table 1 and **Figure 1** show the latitude and longitude of the sampling sites.

Table 2 reports on the detection limits and measured and certified values of element concentrations in the Standard Reference Material (“Trace Metals in Drinking Water” (EU)) that were applied for the evaluation of the precision and validity of the elemental analysis of the filtered water samples. **Table 3** reports on the detection limits and measured and certified values of element concentrations in the Standard Reference Materials (Andesite, AGV-2, and Essexite Gabbro SRM-2A) that were applied for the evaluation of the precision and validity of the elemental analysis of the SPM and sediment samples.

Table 4 shows the distribution of the surface water layer characteristics (Salinity, TSS, DOC, TDN, DOC to TDN ratio (C/N), POC, and POC to DOC ratio (POC/DOC)) along the salinity gradient of the Cai river estuary.

Table 5 reports on the distribution and mean values of the enrichment factor (EF_{Al}) calculated by the double normalization of bulk element to Al in surface SPM along the salinity gradient. **Table 6** reports on the distribution and mean values of the enrichment factor (EF_{Fe}) calculated by the double normalization of bulk element to Fe in surface SPM along the salinity gradient. **Table 7** reports on the distribution and mean values of the enrichment factor (EF_{Al}) calculated by the double normalization of bulk element to Al in surface bottom sediments along the salinity gradient. **Table 8** reports on the distribution and mean values of the enrichment factor (EF_{Fe}) calculated by the double normalization of bulk element to Fe in surface bottom sediments along the salinity gradient.

Tables 9 and 10 show the distribution and mean values of the geoaccumulation index (I_{geo}) of the chemical elements in surface SPM and surface bottom sediments along the salinity gradient.

Tables 11 and 12 show the partitioning coefficient $K_{SPM/Water}$ (or Kd) calculated as the ratio of particulate-to-filtered element concentrations and lg Kd and illustrate the distribution of elements between the dissolved and particulate phases in the surface water layer.

Table 13 reports on the partitioning coefficient $K_{SPM/Sed}$ calculated as the ratio of the element content in surface SPM and surface sediment and illustrates the distribution of elements between the particulate and sedimentary phases.

Table S1 of Supplementary materials covers the source/raw, analysed, and calculated data on the chemical composition, environmental indices and partition coefficients in the solid and solution phases of the Cai river estuary and includes the respective reference material values.

The PCA plot in **Figure 2a** shows the spatial distribution of filtered elements along the salinity gradient.

The PCA plot in **Figure 2b** shows the spatial distribution of the partitioning coefficient $K_{SPM/Water}$ (Kd) along the salinity gradient.

Experimental Design, Materials and Methods

Sampling

The surface water (eight locations, sts. 1–8) and surface sediment samples (seven locations, sts. 2–8) were collected in the Cai River estuary and Nha Trang Bay in July-August 2013 **(Fig. 1)**. The surface water samples were collected using a plastic Niskin Bottle. The salinity and alkalinity were measured on board by portable conductivity apparatuses HI 98129 Combo and HI 98302 DIST 2 (Hanna Instruments, Germany). The suspended particulate matter (SPM) samples were obtained by filtering water samples in an all-glass filtering system. Pre-weighted polycarbonate filters (pore diameter 0.45 µm; Millipore) were used for total suspended matter (TSS), combusted glass fiber filters (GF/F, Whatman) were used for particulate organic carbon (POC), and acid-clean cellulose filters (pore diameter 0.45 µm; Millipore) were used for chemical composition analyses. After sampling, the polycarbonate and cellulose filters were rinsed with 250 ml of Milli-Q water to remove seawater salts. All filters were dried to constant weight at 60° C. After filtration, the filtered water

samples were placed in sterile polypropylene containers, acidified to pH 2 with 65% nitric acid (Merck) for dissolved (filtered) chemical elements (DCE), and with 35% hydrochloric acid (Merck) for dissolved organic carbon (DOC) and total dissolved nitrogen (TDN) analyses and were kept cool until laboratory analyses were performed **[1]**.

The surface sediment samples were collected by scuba divers with a manual plastic piston corer, which was produced at the Shirshov Institute of Oceanology (Russia). The samples were placed into pre-cleaned polyethylene containers using stainless steel spatulas. One portion of the sample was kept frozen until grain size and mineralogy analyses were performed. Another portion was dried to constant weight at 60° C until chemical element analysis was performed.

All sampling, sampling transportation, and preparation procedures were performed using standard clean techniques according to the manual of **[2]**.

Laboratory analyses

The organic geochemistry analyses of the filtered water, SPM and sediment samples were performed at the Ocean Chemistry Laboratory of the Shirshov Institute of Oceanology (RAS). The DOC, POC, TDN, TC and TIC were determined with the analyser TOC 5000-V-CPH (Shimudzu Co., Japan). The dissolved organic carbon (DOC) in the water samples was determined by high-temperature (680 ºC) thermocatalytic oxidation with dispersion-free IP detection. The total dissolved nitrogen (TDN) was determined by catalytic thermal decomposition followed by chemiluminescent detection **[3].** C/N ratios were calculated by dividing DOC by TDN **[1]**. The total carbon (TC) contents in the suspended particulate matter (SPM) and sediment samples were determined by high-temperature (900°C) combustion in airflow using an SSM 5000 A device. The total inorganic carbon (TIC) contents were determined by dry burning at 200º C with H_3PO_4. The total organic carbon (TOC) contents were determined as the difference between TC and TIC contents in the samples. The error of element measurements accounted for 1 rel.%. The reproducibility of the results was within ±5% **[3, 4]**.

Elemental analysis of the filtered water, SPM and sediment samples was performed at the Analytical and Certification Center of the Institute of Microelectronic Technology and High Purity Materials, Russian Academy of Sciences (RAS). Inductively coupled plasma atomic emission spectrometry (ICP-AES) (ICAP-61, Thermo Jarrell Ash, USA) and inductively coupled plasma mass spectrometry (ICP-MS) (X-7, Thermo Elemental, USA) were used for the elemental analysis of the filtered water samples and the solution obtained by the total dissolution of SPM and sediment samples in HNO_3 + $HClO_4$ (3:1 by volume, Merck) in an autoclave system (Ankon-AT-2, Russia). A detailed description of the autoclave digestion procedure is given by **[5]**. ICP-AES was used to measure the major element (Na, Mg, P, S, K, Ca Al, Ti and Fe) and some trace elements (Li, V, Cr, Mn, Co, Ni, Cu, Zn, and Sr); ICP-MS was only used for the analysis of trace elements (Li, Be, B, Sc, Cr, Ni, Cu, Zn, Ga, As, Se, Rb, Sr, Mo, Sn, Sb, Cs, Ba, Tl, Pb, Bi, Th, and U) and rare earth elements (Y, La, Ce,

Pr, Nd, Sm, Eu, Gd, Tb, Dy, Ho, Er, Tm, Yb, and Lu). The combined use of ICP-AES and ICP-MS allows measurements to be validated by comparing the measurements of six elements by both methods simultaneously **[6, 7]**. The error of the element measurements was no greater than 10–15 rel.% for ICP-AES and 10–30 rel.% for ICP-MS, depending on element content.

Accuracy of the analytical determinations

The precision and validity of the analytical determinations were evaluated using Certified Reference Materials (CRM) which were randomly allocated within the determinations. The CRM used included Certified Reference Material "Trace Metals in Drinking Water" (EU) for filtered water samples, Andesite, AGV-2 (United States Geological Survey) and Essexite Gabbro SRM-2A (Russian Geochemical Standard) for SPM and sediment samples. The discrepancy between the certified and measured element contents was within the limits of confidence intervals in every case. The limits of detection were calculated following [6] for all elements (Tables 2 and 3).

Calculation of Enrichment factor and partition coefficients

The Enrichment Factor (EF) normalizes metal contents according to sediment texture properties **[1, 8]**. In the present study, we used both Al and Fe as M_{REF} for the calculation of EF_{Al} and EF_{Fe}, respectively. The EF was calculated as follows:

$$EF=([M]/[M_{REF}]_s)/([M]/[M_{REF}]_b),$$

where the $[M]/[M_{REF}]_s$ is the ratio of the concentrations of the metal to reference metal in the sample, and $[M]/[M_{REF}]_b$ is the ratio of the concentrations of the reference material. Average chemical composition of Suspended Particulate Matter in World Rivers (WRSPM) and average chemical composition of World Shale values were used as a background for SPM and sediments respectively **[9, 10]**.

EF values lower than 1.5 suggest that the element is derived mainly from natural sources, whereas EF values higher than 1.5 suggest anthropogenic sources **[1]**.

The Geoaccumulation Index (I_{geo}) was used to measure metal pollution in sediments and was calculated using the following equation **[3, 8]**:

$$I_{geo} = \log_2(\text{Sample}/1.5\times\text{Background}),$$

where average WRSPM and shale values were used as a background for SPM and sediments, respectively **[9, 10]**. $I_{geo}<0$: uncontaminated; $I_{geo}<1$: uncontaminated to moderately contaminated; $I_{geo}<2$: moderately contaminated; $I_{geo}<3$: moderately to highly contaminated, etc. **[1, 8]**.

The partitioning coefficient ($K_{SPM/Water}$) was calculated as the ratio of element content in surface SPM and filtered water **[1]**. The partitioning coefficient ($K_{SPM/Sed}$) was calculated as the ratio of element content in surface SPM and sediments **[11]**.

Statistical analysis

Descriptive statistics were calculated using Microsoft Excel 2010. Pearson's correlation analysis was performed using SPSS 20.0. Principal Component Analysis (PCA) was conducted using

the MATLAB R2018a computing environment (MathWorks, Inc., USA). RDA analysis was performed in MATLAB R2018a using the Fathom [12]. In all cases, PCA and RDA analyses were carried out on normalized data, i.e. for every variable its mean value was subtracted from the raw data and after that the obtained centered variables were normalized by their standard deviations.

Ethics Statement

The authors declare that they have followed the general ethics rules of scientific research performance and publishing. All applicable international, national, and/or institutional guidelines for the care and use of animals were followed.

CRediT author statement

Koukina Sofia: Conceptualization, Investigation, Methodology, Writing - Original draft preparation; **Lobus Nikolay**: Investigation, Resources, Validation, Writing - Review & Editing; **Shatravin Aleksander**: Software, Data curation, Formal analysis, Visualization

Acknowledgments

This data was collected within the framework of the state assignment of the IO RAS (theme no. 0128-2021-0016).

Declaration of Competing Interest

The authors declare that they have no known competing financial interests or personal relationships which have or could be perceived to have influenced the work reported in this article.

References

[1] S.E. Koukina, N.V. Lobus, A.V. Shatravin **Multi-element signatures in solid and solution phases in a tropical mixing zone: A case study in the Cai River estuary, Vietnam.** Chemosphere, 280 (2021), 130951.

[2] D.H. Loring, R.T.T. Rantala **Manual for the geochemical analyses of marine sediments and suspended particulate matter.** Earth-Science Reviews, 32 (1992), pp. 235–283.

[3] N.V. Lobus, V.I. Peresypkin, N.A. Shulga, A.N. Drozdova, E.S Gesev **Dissolved, particulate, and sedimentary organic matter in the Cai River basin (Nha Trang Bay of the South China Sea).** Oceanology, 55 (2015), pp. 339–346.

[4] S.E. Koukina, N.V. Lobus, V.I. Peresypkin, O.M. Dara, A.V. Smurov **Abundance, distribution and bioavailability of major and trace elements in surface sediments from the Cai River estuary and Nha Trang Bay (South China Sea, Vietnam).** Estuarine, Coastal and Shelf Science, 198 (2017), pp. 450-460.

[5] Y.A. Karpov, V.A. Orlova **Modern methods of autoclave sample preparation in chemical analysis of substances and materials.** Inorganic Materials, 44 (2008), pp. 1501–1508.

[6] V.K. Karandashev, T.A. Orlova, A.E. Lezhnev, S.V. Nosenko, N.I. Zolotareva, I.R. Moskvitina **Use of the inductively coupled plasma mass spectrometry for element analysis of environmental objects.** Inorganic Materials, 44 (2008), pp. 1491–1500.

[7] G.N. Baturin, N.V. Lobus, V.I. Peresypkin, V.T. Komov. **Geochemistry of channel drifts of the Kai River (Vietnam) and sediments of its mouth zone.** Oceanology, 54 (2014), pp. 788-797.
[8] S.E. Koukina, N.V. Lobus **Relationship between enrichment, toxicity, and chemical bioavailability of heavy metals in sediments of the Cai River estuary.** Environmental Monitoring and Assessment, 192 (2020), 305.
[9] K.K. Turekian, K.H. Wedepohl **Distribution of the elements in some major units of the earth's crust.** GSA Bulletin, 72 (1961), pp. 175-192.
[10] J. Viers, B. Dupré, J. Gaillardet **Chemical composition of suspended sediments in World Rivers: New insights from a new database.** Science of the Total Environment, 407 (2009), pp. 853–868.
[11] S. Koukina, N. Lobus **Major and Trace Element Distribution in Suspended Particulate Matter and Sediments of the Tropical River Estuary (South Vietnam)**, in: Chaminé, H.I. (Ed.), Advances in Sustainable and Environmental Hydrology, Hydrogeology, Hydrochemistry and Water Resources, 2019, Springer Nature, Switzerland AG, pp. 65–67.
[12] D.L. Jones **Fathom Toolbox for matlab | USF college of marine science (accessed 4.26.21)**. 2017, https://www.usf.edu/marine-science/research/matlab-resources/fathom-toolbox-for-matlab.aspx

Figure 1. Sampling site locations (Landsat 8 satellite image, 16 August 2013).

Figure 2a. PCA plot for filtered elements.

Figure 2b. PCA plot for partitioning coefficient ($K_{SPM/Water}$).

Table. Location of the sampling sites

Station	Longitude, N	Latitude, E
1	12.271	109.167
2	12.268	109.175
3	12.262	109.197
4	12.261	109.204
5	12.246	109.208
6	12.219	109.225
7	12.198	109.242
8	12.152	109.293

Table 2. Detection limits and measured and certified values of element concentrations in Standard Reference Material

Element	Detection limit				"Trace Metals in Drinking Water" (CRM-TMDW-A-250)[4]	
	Fresh water (< 0.01 ‰)	Brackish water (1-10 ‰)[1]	Brackish water (10-20 ‰)[2]	Sea water (> 20 ‰)[3]	Measured value	Certified value ± SD
**Major elements, mg L^{-1}**						
Na	0.017	0.017	0.017	0.345	5.872	5.936±0.017
Mg	0.008	0.008	0.008	0.153	8.761	8.956±0.018
K	0.01	0.01	0.01	0.193	2.429	2.488±0.009
Ca	0.018	0.018	0.018	0.37	35.06	35.19±0.07
**Trace elements, μg L^{-1}**						
Li	0.003	0.017	0.034	0.137	20.31	20.23±0.12

Be	0.002	0.008	0.016	0.063	20.41	20.33±0.12
B	0.6	3	2	26	< d/l	n.d.
Al	1	3	4	53	121.9	120.0±0.1
Ti	0.7	4	7	28	< d/l	n.d.
V	0.07	0.4	1	3	30.01	30.06±0.02
Cr	0.6	3	6	24	20.24	20.02±0.01
Mn	0.04	0.2	0.4	2	39.77	39.6±0.1
Fe	6	6	15	115	98.99	100.1±0.1
Co	0.08	0.4	1	3	25.34	25.03±0.02
Ni	0.3	0.8	2	7	61.71	60.5±0.5
Cu	0.3	0.6	3	12	20.46	20.03±0.01
Zn	0.4	0.9	4	18	70.73	70.5±0.5
As	0.05	0.2	0.5	2	80.21	80.4±1.0
Se	0.3	1	3	10	10.19	10.13±0.16
Br	8	40	81	323	< d/l	n.d.
Rb	0.006	0.03	0.06	0.24	10.35	10.09±0.07
Zr	0.007	0.033	0.065	0.261	< d/l	n.d.
Nb	0.004	0.022	0.044	0.174	< d/l	n.d.
Sr	0.06	0.3	1	2	246.4	250.3±0.2
Mo	0.016	0.079	0.158	0.632	101.2	99.0±0.7
Ag	0.003	0.017	0.034	0.138	1.99	1.97±0.01
Cd	0.006	0.028	0.055	0.221	10.34	10.01±0.01
Sn	0.012	0.061	0.121	0.485	< d/l	n.d.
Sb	0.008	0.04	0.08	0.323	10.46	9.96±0.14
Te	0.005	0.027	0.054	0.215	2.98	2.95±0.04
Cs	0.005	0.024	0.047	0.189	< d/l	n.d.
Ba	0.03	0.1	0.3	1	49.21	50.7±0.8
W	0.018	0.088	0.176	0.705	< d/l	n.d.
Tl	0.0004	0.002	0.004	0.016	10.0	9.8±0.16
Pb	0.02	0.08	0.2	0.6	39.98	39.3±0.2
Bi	0.0008	0.004	0.004	0.034	9.71	10.01±0.01
Th	0.002	0.011	0.022	0.086	< d/l	n.d.
U	0.0006	0.003	0.006	0.023	10.11	10.01±0.01
**Rare-earth elements, ng L^{-1}**						
Sc	60	300	1000	2000	< d/l	n.d.
Y	1	7	14	57	< d/l	n.d.
La	3	13	26	105	< d/l	n.d.
Ce	2	9	17	68	< d/l	n.d.
Pr	0.4	2	4	15	< d/l	n.d.
Nd	1	5	11	42	< d/l	n.d.
Sm	0.7	3	7	28	< d/l	n.d.
Eu	0.4	2	4	15	< d/l	n.d.
Gd	0.5	3	5	20	< d/l	n.d.
Tb	0.2	1	2	8	< d/l	n.d.
Dy	0.7	4	7	30	< d/l	n.d.
Ho	0.1	0.7	1	5	< d/l	n.d.
Er	0.6	3	6	25	< d/l	n.d.
Tm	0.2	0.8	2	6	< d/l	n.d.
Yb	0.3	1	3	10	< d/l	n.d.
Lu	0.1	0.4	1	3	< d/l	n.d.

Note: n.d. – no data, < d/l – below detection limit; 1 –5 times diluted (Milli-Q); 2 – dilution 10 times; 3 – dilution 40 times; 4 – Matrix: 2% HNO_3 and 0.01% HF.

Table 3. Detection limits and measured and certified values of element concentrations in Standard Reference Materials

Element	Detection limit	Andesite, AGV – 2 (United States Geological Survey)		Essexite Gabbro – 1A (Russian Geochemical Standard, SRM-521-84P)		Black shale SLg – 1 (Russian Geochemical Standard, SRM-8550-04)	
		Measured value	Certified value ± SD	Measured values	Certified value ± SD	Measured values	Certified value ± SD
**Major elements, %**							
Na	0.003	2.75	3.11±0.09	2.08	2.09±0.03	0.98	0.94±0.1
Mg	0.002	1.07	1.08±0.02	4.46	4.22±0.06	-	1.85±0.05
Al	0.002	6.82	8.95±0.11	7.83	7.87±0.04	8.6	8.2±0.16
P	0.004	0.21	0.21±0.01	0.46	0.44±0.01	0.05	0.05±0.004
K	0.003	2.16	2.39±0.09	2.41	2.46±0.041	2.49	2.37±0.09
Ca	0.005	3.43	3.72±0.09	7.79	7.84±0.06	0.8	0.79±0.07
Ti	0.0001	0.58	0.63±0.13	1.0	1.03±0.02	0.52	0.53±0.02
Mn	0.0002	0.062	0.077±0.002	0.12	0.13±0.008	0.085	0.085±0.001
Fe	0.003	4.06	4.68±0.09	8.18	8.16±0.17	5.32	5.23±0.12
**Trace elements, μg g^{-1}**							
Li	0.04	8.81	11*	14.2	14±3	55.3	50±8
Be	0.01	2.1	2.3±0.4	2.2	2.0±	2.0	2.4*
V	2	130	120±5	249	240±20	135	122±15
Cr	1	15.6	17±2	51.7	55±4	122	116±8
Co	0.2	16.8	16±1	45.0	40±5	19.3	20±3
Ni	1	18.6	19±3	38.1	50±5	54.2	50±7
Cu	1	48.5	53±4	61.2	68±7	37.2	39±7
Zn	1	81.0	86±8	122.3	120±10	81.9	97±13
Ga	0.1	22.2	20±1	22	19±2	18.8	18±3
As	0.1	0.5	n.d.	1.8	1.8±0.2	26.3	46±8
Rb	0.1	77.1	68.6±2.3	88.5	73±4	114	112±11
Sr	0.1	672	658±17	2454	2300±200	146	142±15
Zr	0.1	255	230±4	284	240±20	186	176±16
Nb	0.05	14.1	15±1	10.1	8±1	11.4	12±2
Mo	0.08	2.0	n.d.	1.2	1.5±0.5	0.84	0.8*
Ag	0.07	0.091	n.d.	0.11	0.10±0.05	0.5	0.47±0.08
Cd	0.06	0.086	n.d.	< d/l	n.d.	0.3	0.4*
Sn	0.3	2.7	2.3±0.4*	2.7	3.7±0.6*	3	3.2*
Sb	0.05	0.51	0.6*	0.31	n.d.	1.2	1*
Cs	0.01	1.2	1.16±0.08*	3.9	3.8±0.4	4.21	4.0±0.7
Ba	0.08	1160	1140±32	1427	1300±100	378	376±46
Tl	0.003	0.32	0.27*	0.17	n.d.	0.61	n.d.
Pb	0.1	14.0	13±1	20.6	17±2	15.8	15±3
Bi	0.006	0.043	n.d.	0.047	n.d.	0.11	n.d.
Th	0.03	6.6	6.1±0.6	10.1	9±1	8	7.1±1.1
U	0.009	1.9	1.88±0.16	2.8	2.0±0.5	1.7	1.7±0.2
**Rare-earth elements, μg g^{-1}**							
Sc	0.1	12.2	13±1	21.8	27±3	22.5	20±3
Y	0.01	20.0	20±1	33.7	30±4	26	26±4
La	0.02	41.7	38±1	90.7	80±20	28.6	28±5
Ce	0.03	76.1	68±3	204	150±10	59.1	53±8
Pr	0.01	8.4	8.3±0.6	26	15±5	7	6±1
Nd	0.01	33.1	30±2	100	70±10	27.6	25±4
Sm	0.01	5.8	5.7±0.3*	17.6	17±1	5.6	5.4±0.8
Eu	0.01	1.6	1.54±0.1*	4.0	5±1	1.3	1.2±0.2
Gd	0.01	4.9	4.69±0.26	12.3	10±3	5.1	4.5±0.8
Tb	0.003	0.69	0.64±0.04	1.5	1.4±0.2	0.77	0.74*
Dy	0.009	3.8	3.6±0.2	6.9	6±1	4.7	4.4*
Ho	0.007	0.70	0.71±0.08*	1.2	1.2±0.3	0.91	0.92*
Er	0.003	2.0	1.79±0.11*	2.9	3.2±0.7	2.8	2.4*
Tm	0.003	0.27	0.26±0.02	0.36	0.5±0.2	0.37	0.33*
Yb	0.01	1.8	1.6±0.20	2.2	2.9±0.5	2.8	2.7±0.4
Lu	0.01	0.26	0.25±0.01*	0.32	0.3*	0.41	0.4±0.07

* Information values

Table 4. Organic geochemistry parameters of surface water layer

	Stations							
	1	2	3	4	5	6	7	8
Salinity, ‰	< 0.01	3.32	8.49	15.82	24.65	32.85	33.12	33.56
TSS, mg L^{-1}	50.75	41.84	33.12	11.41	4.72	1.35	1.07	1.56
POC, mg L^{-1}	1.18	1.47	1.25	0.94	0.27	0.18	0.18	0.21
DOC, mg L^{-1}	2.42	2.81	2.47	2.33	8.51	4.91	1.22	1.15
DTN, mg L^{-1}	0.48	0.55	0.51	0.49	0.36	0.13	0.11	0.13
DOC/DTN	5.04	5.11	4.84	4.76	23.64	37.77	11.09	8.85
POC/DOC	2.05	1.91	1.98	2.48	31.52	27.28	6.78	5.48
POC, %	2.33	3.51	3.77	8.24	5.72	13.33	16.82	13.46

Table 5. The enrichment factor (EF_{Al}) of elements in surface SPM

Element	Stations								Mean ± SD
	1	2	3	4	5	6	7	8	
**Major elements**									
Mg	0.17	0.21	0.22	0.28	0.64	11.49	13.18	20.94	5.89±8.17
P	0.24	0.28	0.31	0.42	0.61	4.41	4.99	6.20	2.18±2.55
K	0.34	0.37	0.40	0.48	0.73	5.89	6.39	9.29	2.99±1.37
Ca	0.07	0.05	0.03	0.04	0.21	1.85	2.07	3.40	0.96±0.49
Ti	0.23	0.22	0.19	0.16	0.20	0.49	0.41	0.47	0.3±0.14
Mn	0.25	0.17	0.14	0.12	0.21	1.52	1.84	3.19	0.93±1.14
Fe	0.46	0.46	0.46	0.55	0.75	1.06	0.52	0.48	0.59±0.21
**Trace elements**									
Li	3.88	4.05	4.01	4.03	3.45	7.48	7.49	11.42	5.73±2.83
Sc	0.50	0.49	0.48	0.53	0.60	2.92	2.08	2.17	1.22±1.0
V	0.42	0.43	0.48	0.59	0.89	3.43	3.30	1.51	1.38±1.28
Cr	0.83	0.44	0.25	0.33	0.51	3.43	3.41	7.91	2.14±2.69
Co	4.50	3.20	0.34	0.32	0.49	8.37	18.14	58.19	11.7±19.72
Ni	2.98	2.23	0.40	0.49	0.71	7.44	10.76	25.73	6.34±8.67
Cu	1.53	0.88	0.24	0.27	0.36	8.52	10.52	29.66	6.5±10.2
Zn	0.32	0.32	0.33	0.30	0.36	1.00	0.90	1.93	0.68±0.58
Ga	1.22	1.17	1.15	1.17	1.07	2.79	1.03	1.29	1.36±0.58
As	0.67	0.62	0.54	0.65	1.11	5.19	1.22	27.64	4.71±9.4
Rb	0.80	0.78	0.77	0.77	0.90	1.69	0.86	1.16	0.97±0.32
Sr	0.14	0.14	0.14	0.20	0.75	6.32	7.23	12.06	3.37±4.59
Zr	0.10	0.10	0.08	0.08	0.11	0.48	0.18	0.18	0.16±0.13
Nb	0.39	0.36	0.21	0.20	0.26	1.33	0.83	0.97	0.57±0.42
Mo	9.99	8.81	1.73	1.89	2.52	24.99	24.68	50.98	15.7±17.12
Cd	0.11	0.08	0.06	0.01	0.05	0.48	0.00	0.00	0.1±0.16
Sn	1.16	1.02	1.06	0.86	1.14	5.76	3.72	4.33	2.38±1.93
Sb	0.26	0.25	0.24	0.26	0.36	1.85	0.67	1.04	0.62±0.57
Cs	1.50	1.40	1.37	1.13	1.27	2.85	1.04	1.37	1.49±0.57
Ba	0.32	0.28	0.22	0.28	0.42	1.34	0.37	3.39	0.83±1.1
Hf	0.23	0.15	0.14	0.15	0.19	0.69	0.42	0.46	0.3±0.2
W	3.48	2.32	1.60	1.63	2.18	10.20	5.52	5.99	4.12±2.99
Tl	1.06	1.04	0.98	0.99	1.24	1.06	1.09	1.75	1.15±0.26
Pb	0.62	0.61	0.58	0.74	1.15	8.37	4.91	4.92	2.74±2.96
Bi	6.56	7.57	6.55	10.24	15.90	8.72	9.01	5.54	8.76±3.27
Th	2.36	2.31	2.14	1.99	2.51	2.68	2.31	2.35	2.33±0.21
U	1.59	1.77	1.77	1.59	1.50	1.68	0.99	1.45	1.54±0.25
**Rare-earth elements**									

Y	0.98	0.97	0.94	1.18	1.16	3.16	0.85	1.08	1.29±0.76
La	0.89	0.86	0.85	0.85	1.09	1.42	1.03	1.20	1.03±0.21
Ce	1.00	0.98	0.97	0.89	1.21	2.94	1.41	2.93	1.54±0.88
Pr	0.87	0.88	0.85	0.90	0.88	2.51	1.15	1.24	1.16±0.57
Nd	0.80	0.79	0.77	0.82	0.69	1.01	0.60	0.55	0.75±0.14
Sm	0.89	0.86	0.86	0.96	0.86	2.88	1.20	1.32	1.23±0.69
Eu	0.53	0.60	0.53	0.65	0.61	1.06	0.68	0.73	0.67±0.17
Gd	0.94	0.97	0.91	1.06	0.98	1.53	0.87	0.98	1.03±0.21
Tb	0.99	0.97	0.92	1.10	0.80	1.90	0.98	1.25	1.11±0.34
Dy	1.00	1.00	0.99	1.20	0.70	0.86	0.11	0.22	0.76±0.41
Ho	0.96	1.05	0.93	1.14	0.90	1.77	0.90	1.14	1.10±0.29
Er	1.11	1.15	1.05	1.32	1.19	3.10	1.00	1.34	1.41±0.69
Tm	0.92	0.83	0.86	1.08	0.96	1.48	0.94	0.90	1.0±0.21
Yb	1.05	1.07	0.99	1.26	1.22	2.51	0.60	1.04	1.22±0.56
Lu	0.95	0.85	0.90	1.12	0.99	1.96	0.77	1.06	1.07±0.37

Table 6. The enrichment factor (EF_{Fe}) of elements in surface SPM

Element	Stations								Mean ± SD
	1	2	3	4	5	6	7	8	
**Major elements**									
Mg	0.36	0.46	0.47	0.52	0.86	10.88	25.13	43.75	10.3±16.11
Al	2.16	2.15	2.15	1.83	1.33	0.95	1.91	2.09	1.82±0.45
P	0.51	0.60	0.67	0.76	0.82	4.17	9.51	12.95	3.75±4.86
K	0.74	0.80	0.86	0.88	0.97	5.57	12.19	19.42	5.18±7.03
Ca	0.15	0.11	0.07	0.06	0.28	1.75	3.94	7.10	1.68±2.58
Ti	0.50	0.48	0.40	0.30	0.26	0.46	0.78	0.98	0.52±0.24
Mn	0.55	0.37	0.30	0.22	0.27	1.44	3.51	6.68	1.67±2.31
**Trace elements**									
Li	8.35	8.72	8.64	7.39	4.60	7.08	14.29	23.87	10.37±6.1
Sc	1.08	1.06	1.03	0.97	0.80	2.76	3.97	4.53	2.03±1.51
V	0.91	0.92	1.03	1.08	1.18	3.24	6.30	3.15	2.23±1.92
Cr	1.79	0.95	0.54	0.61	0.68	3.25	6.50	16.53	3.86±5.51
Co	9.70	6.89	0.74	0.60	0.65	7.93	34.59	121.61	22.84±41.44
Ni	6.41	4.79	0.86	0.90	0.95	7.04	20.51	53.76	11.9±18.1
Cu	3.30	1.89	0.51	0.49	0.48	8.07	20.06	61.99	12.1±21.23
Zn	0.69	0.70	0.70	0.54	0.48	0.95	1.72	4.03	1.23±1.2
Ga	2.63	2.51	2.49	2.15	1.43	2.64	1.95	2.71	2.31±0.44
As	1.45	1.33	1.17	1.20	1.48	4.91	2.33	57.77	8.96±19.76
Rb	1.72	1.68	1.67	1.41	1.20	1.60	1.63	2.42	1.67±0.35
Sr	0.30	0.30	0.30	0.36	0.99	5.98	13.79	25.20	5.9±9.13
Zr	0.21	0.21	0.17	0.15	0.14	0.46	0.34	0.37	0.26±0.12
Nb	0.84	0.78	0.45	0.36	0.34	1.26	1.58	2.02	0.95±0.62
Mo	21.52	18.96	3.73	3.47	3.36	23.66	47.05	106.53	28.54±34.78
Cd	0.23	0.17	0.12	0.03	0.06	0.46	0.00	0.00	0.13±0.15
Sn	2.50	2.19	2.28	1.57	1.52	5.46	7.08	9.04	3.96±2.87
Sb	0.55	0.54	0.51	0.47	0.48	1.75	1.28	2.18	0.97±0.68
Cs	3.24	3.01	2.95	2.07	1.70	2.70	1.99	2.87	2.56±0.57
Ba	0.68	0.61	0.48	0.52	0.57	1.27	0.70	7.08	1.49±2.27
Hf	0.50	0.32	0.29	0.28	0.25	0.66	0.81	0.96	0.51±0.27
W	7.51	4.99	3.44	2.99	2.91	9.66	10.53	12.52	6.82±3.77
Tl	2.28	2.23	2.10	1.81	1.65	1.00	2.07	3.66	2.1±0.75
Pb	1.33	1.31	1.26	1.35	1.54	7.92	9.36	10.29	4.29±4.11
Bi	14.14	16.29	14.11	18.78	21.21	8.25	17.17	11.58	15.19±4.1
Th	5.08	4.98	4.61	3.65	3.34	2.54	4.40	4.92	4.19±0.92
U	3.42	3.81	3.81	2.91	2.00	1.59	1.89	3.03	2.81±0.88
**Rare-earth elements**									

Y	2.12	2.08	2.03	2.17	1.55	2.99	1.61	2.26	2.1±0.44
La	1.93	1.85	1.83	1.57	1.45	1.35	1.97	2.52	1.81±0.37
Ce	2.15	2.11	2.09	1.64	1.62	2.78	2.69	6.11	2.65±1.46
Pr	1.88	1.88	1.83	1.65	1.18	2.38	2.20	2.59	1.95±0.44
Nd	1.71	1.70	1.65	1.50	0.93	0.96	1.15	1.14	1.34±0.34
Sm	1.91	1.84	1.85	1.76	1.14	2.73	2.29	2.75	2.03±0.54
Eu	1.14	1.29	1.14	1.19	0.82	1.01	1.29	1.52	1.17±0.21
Gd	2.02	2.08	1.96	1.94	1.30	1.45	1.66	2.04	1.81±0.3
Tb	2.12	2.09	1.98	2.02	1.07	1.80	1.88	2.61	1.94±0.43
Dy	2.15	2.16	2.13	2.21	0.93	0.82	0.21	0.46	1.38±0.86
Ho	2.07	2.26	2.01	2.10	1.20	1.67	1.72	2.39	1.93±0.38
Er	2.40	2.48	2.27	2.42	1.58	2.93	1.90	2.81	2.35±0.44
Tm	1.98	1.78	1.86	1.98	1.28	1.40	1.80	1.87	1.74±0.26
Yb	2.27	2.30	2.13	2.32	1.62	2.37	1.14	2.17	2.04±0.43
Lu	2.05	1.83	1.93	2.06	1.32	1.85	1.46	2.22	1.84±0.31

Table 7. The enrichment factor (EF_{Al}) of elements in surface sediments

Element	Stations							Mean ± SD
	2	3	4	5	6	7	8	
**Major elements**								
P	0.54	0.46	0.40	0.44	0.43	0.59	0.66	0.5±0.1
S	0.76	0.80	0.94	1.79	1.37	2.58	0.90	1.31±0.67
K	0.39	0.37	0.73	0.48	0.39	0.66	0.68	0.53±0.16
Ca	0.13	0.15	0.22	0.25	0.22	4.02	1.88	0.98±1.48
Ti	0.56	0.50	0.54	0.60	0.53	0.78	0.81	0.62±0.13
Mn	0.49	0.28	0.50	0.31	0.22	0.54	0.64	0.43±0.15
Fe	0.63	0.60	0.52	0.62	0.55	0.81	0.83	0.65±0.12
**Trace elements**								
Li	0.44	0.43	0.53	0.50	0.46	0.95	0.89	0.6±0.22
Be	0.63	0.56	0.61	0.61	0.62	0.70	0.71	0.64±0.05
Sc	0.77	0.71	0.63	0.79	0.70	0.88	0.91	0.77±0.1
V	0.50	0.48	0.48	0.47	0.45	0.66	0.67	0.53±0.09
Cr	0.33	0.33	0.31	0.31	0.29	0.62	0.70	0.41±0.17
Co	0.32	0.26	0.26	0.32	0.28	0.49	0.60	0.36±0.13
Ni	0.23	0.21	0.21	0.20	0.20	0.42	0.53	0.29±0.13
Cu	0.32	0.29	0.30	0.26	0.28	0.28	0.38	0.3±0.04
Zn	0.81	0.76	0.74	0.80	0.80	0.97	0.99	0.84±0.1
Ga	1.01	0.97	0.92	0.98	0.97	0.97	0.94	0.97±0.03
As	1.54	1.41	1.15	1.17	1.00	1.47	0.88	1.23±0.25
Rb	0.68	0.63	1.07	0.77	0.69	1.06	1.04	0.85±0.2
Sr	0.14	0.14	0.21	0.18	0.17	2.10	0.91	0.55±0.74
Zr	0.36	0.33	0.39	0.44	0.35	0.55	0.44	0.41±0.08
Nb	1.15	1.04	1.10	1.18	1.17	1.27	1.31	1.17±0.09
Mo	1.27	0.88	0.81	0.72	0.95	0.23	0.18	0.72±0.39
Ag	0.88	0.66	0.00	0.87	0.83	0.00	0.00	0.46±0.44
Cd	0.00	0.31	0.00	0.28	0.33	0.00	0.00	0.13±0.16
Sn	0.08	0.07	0.07	0.07	0.06	0.06	0.09	0.07±0.01
Sb	0.57	0.56	0.57	0.61	0.49	0.71	0.68	0.6±0.08
Cs	1.71	1.55	1.59	1.56	1.55	1.91	1.79	1.67±0.14
Ba	0.31	0.28	0.48	0.31	0.29	0.51	0.52	0.39±0.11
Hf	0.65	0.63	0.87	0.83	0.81	0.96	0.72	0.78±0.12
W	0.75	0.65	0.73	0.71	0.71	0.71	0.62	0.7±0.05
Hg	0.20	0.16	0.20	0.17	0.08	0.13	0.14	0.15±0.04
Tl	0.49	0.45	0.64	0.52	0.51	0.61	0.55	0.54±0.07
Pb	1.98	1.87	1.98	2.03	2.19	1.83	2.07	2.0±0.12
Bi	34.33	22.53	21.49	3.08	3.40	1.96	2.05	12.69±13.22

Th	2.38	2.21	2.01	2.22	2.29	2.05	1.71	2.12±0.23
U	1.41	1.13	1.22	1.42	1.38	1.49	0.98	1.29±0.18
**Rare-earth elements**								
Y	0.87	0.74	0.75	0.94	0.90	0.75	0.65	0.8±0.1
La	1.06	0.92	1.20	1.17	1.12	1.60	1.50	1.23±0.23
Ce	1.11	1.02	1.29	1.24	1.24	1.67	1.52	1.3±0.23
Pr	0.97	0.85	1.11	1.10	1.04	1.44	1.33	1.12±0.2
Nd	0.97	0.84	1.07	1.11	1.01	1.40	1.30	1.1±0.19
Sm	1.07	0.92	1.05	1.23	1.13	1.38	1.28	1.15±0.15
Eu	0.68	0.58	0.72	0.76	0.70	1.05	1.08	0.8±0.19
Gd	1.04	0.88	0.94	1.16	1.06	1.16	1.04	1.04±0.11
Tb	0.93	0.80	0.83	1.00	1.00	1.02	0.91	0.93±0.09
Dy	1.13	0.97	1.00	1.21	1.17	1.09	0.96	1.08±0.1
Ho	0.86	0.74	0.76	0.92	0.92	0.80	0.70	0.81±0.09
Er	0.76	0.65	0.66	0.81	0.79	0.69	0.60	0.71±0.08
Tm	0.74	0.64	0.68	0.78	0.77	0.68	0.62	0.7±0.06
Yb	0.80	0.70	0.72	0.86	0.83	0.73	0.62	0.75±0.08
Lu	0.73	0.64	0.66	0.81	0.78	0.64	0.58	0.69±0.08

Average Chemical Composition of Ocean Shale by Turekian and Wedepohl (1961) (1).

Table 8. The enrichment factor (EF_{Fe}) of elements in surface sediments

Element	Stations							Mean ± SD
	2	3	4	5	6	7	8	
**Major elements**								
Al	1.60	1.67	1.90	1.62	1.83	1.24	1.21	1.58±0.27
P	0.86	0.76	0.76	0.72	0.79	0.72	0.80	0.77±0.05
S	1.22	1.34	1.79	2.90	2.51	3.19	1.09	2.01±0.86
K	0.62	0.61	1.39	0.77	0.71	0.82	0.83	0.82±0.27
Ca	0.21	0.25	0.42	0.40	0.39	4.97	2.27	1.27±1.79
Ti	0.89	0.84	1.03	0.98	0.96	0.97	0.98	0.95±0.06
Mn	0.79	0.47	0.95	0.50	0.41	0.67	0.77	0.65±0.2
**Trace elements**								
Li	0.71	0.72	1.01	0.82	0.84	1.17	1.08	0.91±0.18
Be	1.01	0.94	1.17	1.00	1.13	0.87	0.86	1.0±0.12
Sc	1.22	1.18	1.20	1.28	1.28	1.09	1.10	1.19±0.08
V	0.80	0.80	0.91	0.76	0.82	0.82	0.81	0.82±0.04
Cr	0.53	0.55	0.59	0.51	0.53	0.77	0.84	0.62±0.13
Co	0.52	0.43	0.50	0.52	0.51	0.61	0.73	0.54±0.1
Ni	0.37	0.36	0.40	0.33	0.36	0.52	0.64	0.42±0.11
Cu	0.51	0.49	0.57	0.41	0.51	0.35	0.46	0.47±0.07
Zn	1.29	1.26	1.41	1.29	1.45	1.20	1.20	1.3±0.1
Ga	1.62	1.63	1.75	1.60	1.77	1.19	1.14	1.53±0.26
As	2.46	2.36	2.18	1.90	1.82	1.82	1.07	1.94±0.47
Rb	1.08	1.05	2.03	1.25	1.27	1.31	1.25	1.32±0.33
Sr	0.23	0.24	0.39	0.30	0.31	2.60	1.10	0.74±0.88
Zr	0.57	0.55	0.74	0.71	0.64	0.69	0.53	0.63±0.08
Nb	1.83	1.73	2.10	1.91	2.14	1.57	1.58	1.84±0.23
Mo	2.04	1.47	1.55	1.16	1.73	0.29	0.22	1.21±0.7
Ag	1.40	1.10	0.00	1.41	1.51	0.00	0.00	0.77±0.74
Cd	0.00	0.51	0.00	0.45	0.60	0.00	0.00	0.22±0.28
Sn	0.13	0.11	0.13	0.11	0.12	0.08	0.10	0.11±0.02
Sb	0.92	0.94	1.09	1.00	0.89	0.88	0.82	0.93±0.09
Cs	2.73	2.60	3.03	2.53	2.83	2.36	2.16	2.61±0.29
Ba	0.49	0.46	0.90	0.51	0.52	0.64	0.63	0.59±0.15
Hf	1.05	1.06	1.66	1.34	1.48	1.18	0.87	1.23±0.28
W	1.20	1.09	1.39	1.14	1.30	0.88	0.75	1.11±0.23
Hg	0.31	0.27	0.37	0.27	0.15	0.16	0.17	0.24±0.09
Tl	0.78	0.76	1.22	0.85	0.92	0.75	0.66	0.85±0.18
Pb	3.17	3.14	3.78	3.29	4.01	2.26	2.51	3.16±0.63
Bi	54.91	37.70	40.94	5.00	6.20	2.43	2.48	21.38±22.31

Th	3.81	3.70	3.82	3.60	4.18	2.54	2.06	3.39±0.78
U	2.26	1.88	2.32	2.30	2.52	1.84	1.19	2.04±0.45
**Rare-earth elements**								
Y	1.39	1.23	1.44	1.52	1.65	0.93	0.79	1.28±0.32
La	1.69	1.55	2.30	1.89	2.04	1.99	1.82	1.9±0.24
Ce	1.77	1.71	2.46	2.00	2.27	2.07	1.83	2.02±0.27
Pr	1.56	1.42	2.11	1.78	1.90	1.79	1.60	1.74±0.23
Nd	1.54	1.41	2.03	1.81	1.85	1.73	1.57	1.71±0.21
Sm	1.71	1.54	2.00	1.99	2.06	1.70	1.54	1.79±0.22
Eu	1.09	0.97	1.37	1.24	1.28	1.30	1.30	1.22±0.14
Gd	1.66	1.47	1.79	1.89	1.93	1.44	1.26	1.63±0.25
Tb	1.49	1.35	1.58	1.62	1.83	1.27	1.10	1.46±0.24
Dy	1.81	1.62	1.90	1.96	2.14	1.35	1.16	1.71±0.35
Ho	1.37	1.25	1.45	1.50	1.67	0.99	0.85	1.3±0.29
Er	1.21	1.09	1.26	1.31	1.45	0.85	0.73	1.13±0.26
Tm	1.19	1.07	1.29	1.26	1.41	0.84	0.75	1.12±0.24
Yb	1.28	1.18	1.37	1.39	1.52	0.91	0.75	1.2±0.28
Lu	1.17	1.07	1.27	1.31	1.43	0.80	0.70	1.11±0.27

Average Chemical Composition of Ocean Shale by Turekian and Wedepohl (1961)

Table 9. Geoaccumulation Index (I_{geo}) of elements in surface SPM

Element	Stations								Mean
	1	2	3	4	5	6	7	8	
**Major elements**									
Mg	-2,36	-2,01	-1,94	-1,65	-1,05	0,30	0,39	0,32	-1,00
Al	0,21	0,22	0,24	0,16	-0,41	-3,22	-3,33	-4,07	-1,28
P	-1,86	-1,61	-1,45	-1,10	-1,12	-1,08	-1,01	-1,44	-1,34
K	-1,34	-1,21	-1,08	-0,91	-0,87	-0,67	-0,65	-0,85	-0,95
Ca	-3,68	-4,06	-4,62	-4,66	-2,67	-2,34	-2,28	-2,30	-3,33
Ti	-1,91	-1,96	-2,18	-2,44	-2,75	-4,26	-4,61	-5,17	-3,16
Mn	-1,77	-2,34	-2,62	-2,88	-2,70	-2,62	-2,45	-2,39	-2,47
Fe	-0,90	-0,89	-0,87	-0,71	-0,83	-3,14	-4,26	-5,13	-2,09
**Trace elements**									
Li	2,16	2,24	2,24	2,17	1,37	-0,32	-0,42	-0,56	1,11
Sc	-0,78	-0,80	-0,82	-0,76	-1,14	-1,68	-2,27	-2,95	-1,40
V	-1,04	-1,01	-0,82	-0,60	-0,59	-1,45	-1,61	-3,48	-1,32
Cr	-0,06	-0,96	-1,77	-1,44	-1,38	-1,45	-1,56	-1,09	-1,21
Co	2,38	1,90	-1,30	-1,46	-1,46	-0,16	0,85	1,79	0,32
Ni	1,78	1,37	-1,09	-0,86	-0,91	-0,33	0,10	0,62	0,08
Cu	0,82	0,03	-1,85	-1,73	-1,88	-0,13	0,07	0,82	-0,48
Zn	-1,43	-1,40	-1,38	-1,59	-1,89	-3,22	-3,48	-3,12	-2,19
Ga	0,50	0,44	0,44	0,39	-0,31	-1,74	-3,29	-3,70	-0,91
As	-0,36	-0,48	-0,64	-0,45	-0,26	-0,85	-3,04	0,72	-0,67
Rb	-0,12	-0,14	-0,13	-0,22	-0,57	-2,47	-3,55	-3,86	-1,38
Sr	-2,63	-2,61	-2,59	-2,19	-0,84	-0,56	-0,47	-0,48	-1,55
Zr	-3,13	-3,17	-3,40	-3,41	-3,66	-4,28	-5,82	-6,57	-4,18
Nb	-1,15	-1,25	-2,03	-2,17	-2,38	-2,81	-3,60	-4,12	-2,44
Mo	3,53	3,36	1,03	1,08	0,92	1,42	1,30	1,60	1,78
Cd	-3,02	-3,47	-3,88	-5,99	-4,86	-4,28	-	-	-4,25
Sn	0,42	0,24	0,32	-0,06	-0,23	-0,70	-1,44	-1,96	-0,42
Sb	-1,76	-1,77	-1,83	-1,81	-1,88	-2,34	-3,91	-4,01	-2,41
Cs	0,80	0,70	0,69	0,33	-0,07	-1,71	-3,27	-3,61	-0,77
Ba	-1,45	-1,61	-1,93	-1,66	-1,65	-2,80	-4,78	-2,31	-2,27
Hf	-1,90	-2,53	-2,64	-2,57	-2,83	-3,75	-4,57	-5,19	-3,25
W	2,01	1,43	0,91	0,86	0,71	0,13	-0,86	-1,49	0,46
Tl	0,29	0,27	0,20	0,14	-0,10	-3,14	-3,21	-3,26	-1,10
Pb	-0,49	-0,50	-0,54	-0,28	-0,21	-0,16	-1,03	-1,77	-0,62
Bi	2,92	3,14	2,95	3,52	3,58	-0,10	-0,16	-1,60	1,78
Th	1,45	1,43	1,34	1,15	0,91	-1,80	-2,12	-2,84	-0,06
U	0,87	1,04	1,06	0,83	0,17	-2,48	-3,34	-3,53	-0,67
**Rare-earth elements**									
Y	0,18	0,17	0,15	0,40	-0,20	-1,57	-3,57	-3,96	-1,05
La	0,05	0,00	0,00	-0,07	-0,29	-2,72	-3,28	-3,80	-1,26
Ce	0,20	0,19	0,19	-0,01	-0,14	-1,67	-2,83	-2,52	-0,82
Pr	0,01	0,03	0,00	0,01	-0,60	-1,89	-3,12	-3,76	-1,17
Nd	-0,12	-0,12	-0,15	-0,13	-0,94	-3,21	-4,06	-4,95	-1,71
Sm	0,03	-0,01	0,02	0,10	-0,64	-1,70	-3,06	-3,67	-1,12
Eu	-0,71	-0,52	-0,68	-0,47	-1,12	-3,14	-3,89	-4,53	-1,88
Gd	0,12	0,17	0,10	0,24	-0,45	-2,61	-3,53	-4,10	-1,26
Tb	0,19	0,18	0,12	0,30	-0,73	-2,30	-3,35	-3,75	-1,17
Dy	0,20	0,22	0,22	0,43	-0,93	-3,43	-6,53	-6,26	-2,01
Ho	0,15	0,29	0,13	0,35	-0,57	-2,40	-3,48	-3,88	-1,17
Er	0,36	0,42	0,31	0,56	-0,17	-1,59	-3,33	-3,64	-0,88
Tm	0,09	-0,05	0,03	0,27	-0,48	-2,66	-3,41	-4,23	-1,31
Yb	0,28	0,31	0,22	0,50	-0,13	-1,90	-4,07	-4,01	-1,10
Lu	0,14	-0,01	0,08	0,33	-0,43	-2,25	-3,71	-3,98	-1,23

Table 10. Geoaccumulation Index (I_{geo}) of elements in surface sediments

Element	Stations							Mean
	2	3	4	5	6	7	8	
**Major elements**								
Al	0,03	0,10	-0,59	-0,02	0,12	-0,64	-0,50	-0,22
P	-0,87	-1,03	-1,93	-1,20	-1,10	-1,42	-1,10	-1,24
S	-0,36	-0,22	-0,68	0,82	0,58	0,72	-0,65	0,03
K	-1,34	-1,35	-1,05	-1,09	-1,24	-1,24	-1,05	-1,19
Ca	-2,93	-2,64	-2,77	-2,03	-2,09	1,36	0,41	-1,53
Ti	-0,82	-0,90	-1,48	-0,75	-0,81	-1,00	-0,79	-0,94
Mn	-1,00	-1,73	-1,61	-1,73	-2,04	-1,53	-1,14	-1,54
Fe	-0,65	-0,64	-1,52	-0,72	-0,75	-0,95	-0,77	-0,86
**Trace elements**								
Li	-1,14	-1,12	-1,51	-1,01	-0,99	-0,72	-0,66	-1,02
Be	-0,64	-0,73	-1,30	-0,72	-0,57	-1,16	-0,98	-0,87
Sc	-0,36	-0,41	-1,26	-0,37	-0,39	-0,82	-0,64	-0,61
V	-0,97	-0,96	-1,67	-1,12	-1,03	-1,25	-1,08	-1,15
Cr	-1,56	-1,50	-2,27	-1,71	-1,66	-1,32	-1,02	-1,58
Co	-1,60	-1,87	-2,53	-1,67	-1,72	-1,67	-1,23	-1,75
Ni	-2,10	-2,12	-2,84	-2,33	-2,23	-1,91	-1,42	-2,14
Cu	-1,63	-1,68	-2,33	-1,99	-1,73	-2,48	-1,89	-1,96
Zn	-0,28	-0,31	-1,03	-0,35	-0,21	-0,69	-0,51	-0,48
Ga	0,05	0,06	-0,71	-0,04	0,07	-0,70	-0,58	-0,27
As	0,65	0,59	-0,40	0,20	0,12	-0,09	-0,68	0,06
Rb	-0,54	-0,57	-0,50	-0,40	-0,41	-0,56	-0,44	-0,49
Sr	-2,76	-2,70	-2,88	-2,47	-2,45	0,43	-0,64	-1,93
Zr	-1,45	-1,51	-1,96	-1,21	-1,39	-1,49	-1,69	-1,53
Nb	0,23	0,15	-0,45	0,21	0,35	-0,30	-0,11	0,01
Mo	0,38	-0,09	-0,89	-0,51	0,05	-2,76	-2,94	-0,97
Ag	-0,16	-0,50	-	-0,23	-0,15	-	-	-0,26
Cd	-	-1,61	-	-1,87	-1,48	-	-	-1,65
Sn	-3,56	-3,79	-4,44	-3,91	-3,85	-4,59	-4,05	-4,03
Sb	-0,77	-0,73	-1,40	-0,73	-0,92	-1,13	-1,06	-0,96
Cs	0,80	0,73	0,08	0,62	0,75	0,29	0,34	0,52
Ba	-1,68	-1,76	-1,67	-1,69	-1,69	-1,60	-1,43	-1,65
Hf	-0,58	-0,56	-0,79	-0,30	-0,18	-0,71	-0,98	-0,59
W	-0,38	-0,51	-1,05	-0,53	-0,37	-1,14	-1,19	-0,74
Hg	-2,33	-2,54	-2,94	-2,62	-3,46	-3,64	-3,30	-2,97
Tl	-1,01	-1,04	-1,24	-0,96	-0,86	-1,37	-1,36	-1,12
Pb	1,02	1,00	0,39	1,00	1,25	0,23	0,56	0,78
Bi	5,13	4,59	3,83	1,60	1,88	0,33	0,54	2,56
Th	1,28	1,24	0,41	1,13	1,32	0,39	0,28	0,86
U	0,53	0,27	-0,31	0,48	0,58	-0,07	-0,52	0,14
**Rare-earth elements**								
Y	-0,17	-0,34	-1,00	-0,11	-0,03	-1,05	-1,11	-0,55
La	0,11	-0,02	-0,33	0,20	0,28	0,04	0,09	0,05
Ce	0,18	0,13	-0,23	0,28	0,43	0,10	0,11	0,14
Pr	-0,01	-0,14	-0,45	0,12	0,18	-0,12	-0,09	-0,07
Nd	-0,02	-0,15	-0,50	0,13	0,14	-0,16	-0,12	-0,10
Sm	0,13	-0,02	-0,52	0,27	0,29	-0,18	-0,14	-0,03
Eu	-0,53	-0,69	-1,07	-0,41	-0,39	-0,57	-0,39	-0,58
Gd	0,08	-0,09	-0,68	0,20	0,20	-0,43	-0,44	-0,17
Tb	-0,07	-0,22	-0,87	-0,03	0,12	-0,61	-0,64	-0,33
Dy	0,21	0,05	-0,60	0,25	0,35	-0,52	-0,56	-0,12

Ho	-0,20	-0,33	-0,99	-0,14	-0,01	-0,96	-1,01	-0,52
Er	-0,38	-0,52	-1,19	-0,33	-0,21	-1,19	-1,23	-0,72
Tm	-0,40	-0,55	-1,16	-0,38	-0,25	-1,21	-1,18	-0,73
Yb	-0,30	-0,41	-1,07	-0,25	-0,15	-1,09	-1,19	-0,64
Lu	-0,42	-0,54	-1,18	-0,33	-0,23	-1,28	-1,28	-0,75

Table 11. The partitioning coefficient ($K_{SPM/Water}$)

Element	Stations (Salinity, ‰)				
	1 (< 0.01)	3 (8.49)	4 (15.82)	7 (33.12)	8 (33.56)
Li	56565	6158	2000	57	55
Na	92	11	8	23	22
Mg	2551	64	25	16	16
K	5612	520	191	34	31
Ca	865	47	17	17	18
Rb	16662	8036	3764	82	71
Sr	1716	103	49	28	30
Mo	261645	9894	4391	982	1245
Cs	169390	80156	59998	3313	2861
Ba	13628	7401	10664	4277	22607
U	86535	50063	16877	124	116

Table 12. The partitioning coefficient (lg ($K_{SPM/Water}$))

Element	Stations (Salinity, ‰)				
	1 (< 0.01)	3 (8.49)	4 (15.82)	7 (33.12)	8 (33.56)
Li	4.75	3.79	3.30	1.75	1.74
Na	1.96	1.05	0.91	1.37	1.34
Mg	3.41	1.81	1.39	1.21	1.21
K	3.75	2.72	2.28	1.53	1.49
Ca	2.94	1.67	1.23	1.23	1.26
Rb	4.22	3.91	3.58	1.91	1.85
Sr	3.23	2.01	1.69	1.44	1.47
Mo	5.42	4.00	3.64	2.99	3.10
Cs	5.23	4.90	4.78	3.52	3.46
Ba	4.13	3.87	4.03	3.63	4.35
U	4.94	4.70	4.23	2.09	2.07

Table 13. The partitioning coefficient ($K_{SPM/Sed}$)

Element	Stations							Mean ± SD
	2	3	4	5	6	7	8	
**Major elements**								
Al	1.24	1.20	1.84	0.83	0.11	0.17	0.09	0.78±0.68
P	1.72	2.15	5.07	3.04	2.89	3.80	2.27	2.99±1.14
K	0.70	0.76	0.70	0.74	0.95	0.95	0.73	0.79±0.11
Ca	0.76	0.43	0.45	1.07	1.42	0.13	0.26	0.65±0.46
Ti	0.43	0.39	0.49	0.24	0.09	0.08	0.05	0.25±0.19

Mn	0.78	1.07	0.82	1.01	1.32	1.05	0.83	0.98±0.19
Fe	1.04	1.05	2.16	1.14	0.23	0.12	0.06	0.83±0.75
**Trace elements**								
Li	1.34	1.33	1.65	0.67	0.21	0.16	0.14	0.78±0.65
Be	1.26	1.30	1.64	1.05	0.50	0.09	0.06	0.84±0.63
Sc	1.03	1.05	1.98	0.82	0.57	0.51	0.28	0.89±0.56
V	0.97	1.09	2.08	1.44	0.75	0.77	0.19	1.04±0.6
Cr	2.19	1.20	2.58	1.81	1.67	1.23	1.38	1.72±0.52
Co	13.40	1.75	2.48	1.37	3.49	6.78	9.61	5.55±4.57
Ni	12.16	2.24	4.33	2.94	4.08	4.40	4.50	4.95±3.29
Cu	5.34	1.50	2.55	1.83	5.09	9.86	11.05	5.32±3.83
Zn	1.01	1.04	1.48	0.76	0.27	0.32	0.36	0.75±0.46
Ga	1.25	1.24	2.05	0.79	0.27	0.16	0.11	0.84±0.72
As	1.28	1.19	2.69	2.02	1.43	0.36	7.35	2.33±2.33
Rb	0.74	0.76	0.68	0.50	0.13	0.07	0.05	0.42±0.32
Sr	0.69	0.67	1.00	1.93	2.31	0.33	0.70	1.09±0.74
Zr	0.30	0.27	0.37	0.18	0.13	0.05	0.03	0.19±0.13
Nb	0.44	0.27	0.37	0.20	0.14	0.13	0.08	0.23±0.14
Mo	9.06	2.48	4.49	3.08	2.97	19.09	26.72	9.7±9.54
Ag	2.45	2.38	-	2.34	2.01	-	-	-
Cd	-	1.07	-	0.65	0.74	-	-	-
Sn	1.07	1.32	1.59	0.98	0.68	0.68	0.32	0.95±0.43
Sb	0.73	0.68	1.10	0.65	0.55	0.21	0.19	0.59±0.32
Cs	1.17	1.21	1.49	0.78	0.23	0.11	0.08	0.72±0.59
Ba	0.94	0.80	0.91	0.93	0.42	0.10	0.49	0.65±0.32
Hf	0.37	0.34	0.42	0.25	0.12	0.10	0.08	0.24±0.14
W	3.88	2.98	4.18	2.61	1.56	1.34	0.90	2.49±1.27
Tl	0.92	0.90	0.99	0.69	0.08	0.11	0.10	0.54±0.43
Pb	1.07	1.05	1.91	1.33	1.15	1.27	0.61	1.2±0.39
Bi	0.50	0.63	1.59	7.77	0.50	1.41	0.45	1.83±2.66
Th	1.12	1.08	1.69	0.87	0.12	0.18	0.12	0.74±0.61
U	1.28	1.54	1.96	0.72	0.11	0.09	0.11	0.83±0.77
**Rare-earth elements**								
Y	1.07	1.19	2.23	0.79	0.29	0.15	0.12	0.83±0.75
La	1.08	1.18	1.40	0.83	0.15	0.12	0.08	0.69±0.56
Ce	1.06	1.10	1.23	0.79	0.24	0.14	0.17	0.67±0.48
Pr	1.03	1.11	1.38	0.61	0.24	0.13	0.08	0.65±0.53
Nd	0.97	1.04	1.34	0.49	0.10	0.07	0.04	0.58±0.54
Sm	0.98	1.10	1.65	0.57	0.27	0.15	0.09	0.69±0.58
Eu	1.08	1.09	1.63	0.66	0.16	0.11	0.06	0.68±0.61
Gd	1.08	1.15	1.91	0.65	0.14	0.12	0.08	0.73±0.69
Tb	1.15	1.22	2.16	0.59	0.18	0.14	0.11	0.79±0.76
Dy	1.07	1.20	2.17	0.47	0.08	0.02	0.02	0.72±0.81
Ho	1.18	1.17	2.15	0.63	0.16	0.15	0.12	0.79±0.76
Er	1.14	1.17	2.21	0.73	0.25	0.15	0.12	0.83±0.75
Tm	0.97	1.13	2.04	0.71	0.14	0.16	0.09	0.75±0.71
Yb	1.04	1.05	2.01	0.74	0.20	0.09	0.10	0.75±0.7
Lu	0.97	1.12	2.08	0.68	0.18	0.14	0.11	0.75±0.71

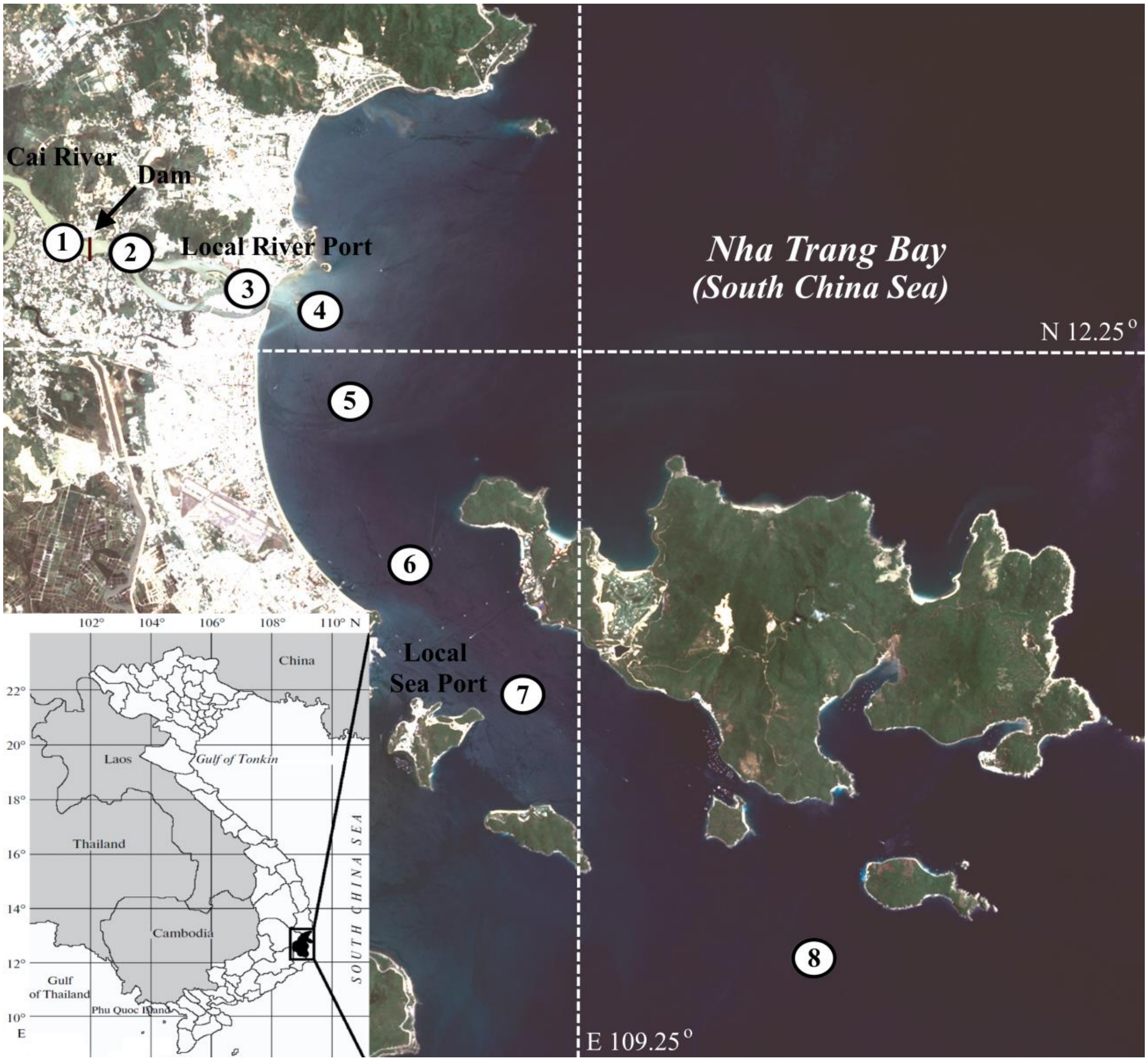
Cai River
Dam
1
2
Local River Port
3
4
Nha Trang Bay
(South China Sea)
N 12.25°
5
6
Local
Sea Port
7
8
E 109.25°
102°
104°
106°
108°
110° N
China
22°
20°
Laos
Gulf of Tonkin
18°
Thailand
16°
14°
Cambodia
12°
Gulf
of Thailand
Phu Quoc Island
10°
E
SOUTH CHINA SEA

Fig. 1

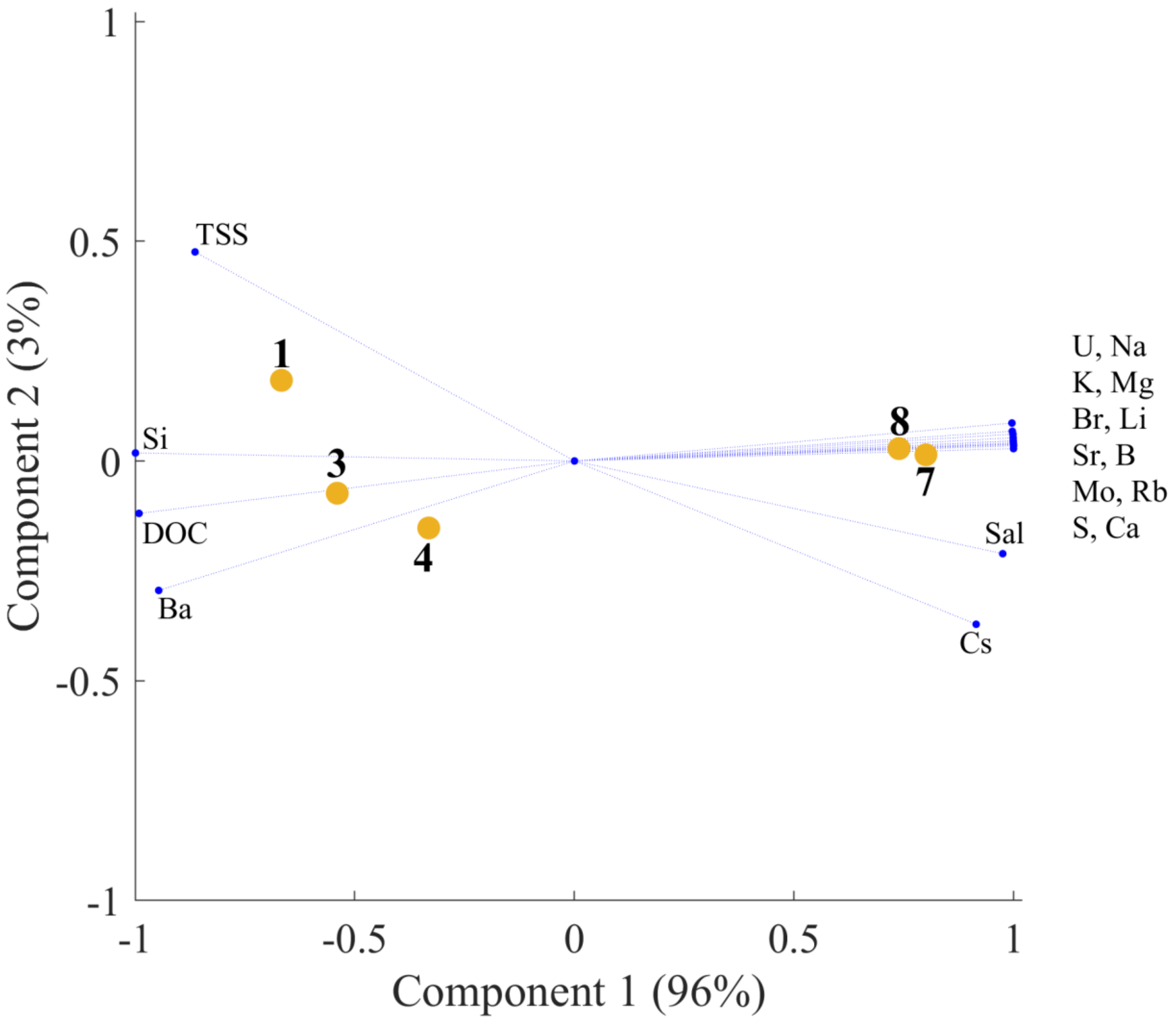
1
0.5
0
-0.5
-1
-1
-0.5
0
0.5
1
Component 2 (3%)
Component 1 (96%)
TSS
Si
DOC
Ba
1
3
4
8
7
Sal
Cs
U, Na
K, Mg
Br, Li
Sr, B
Mo, Rb
S, Ca

Fig. 2 (a)

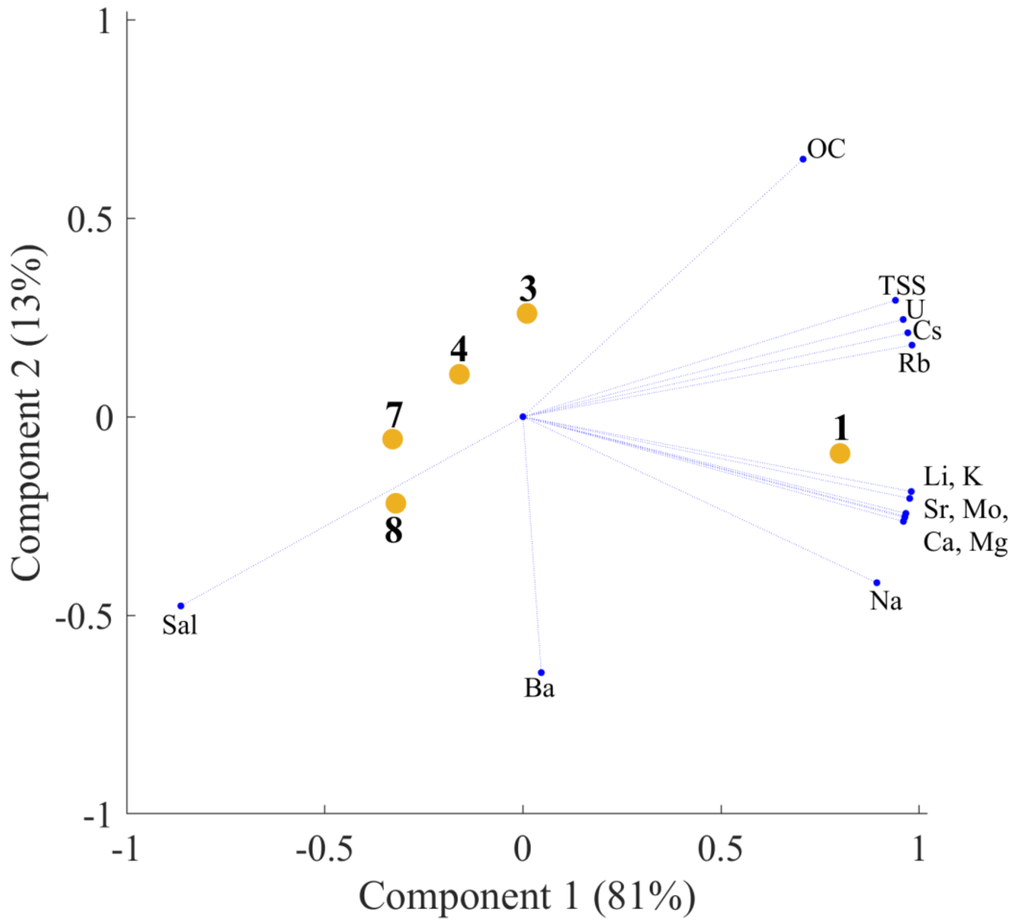
1
0.5
0
-0.5
-1
-1
-0.5
0
0.5
1
Component 2 (13%)
Component 1 (81%)
OC
TSS
U
Cs
Rb
Li, K
Sr, Mo,
Ca, Mg
Na
Ba
Sal
1
3
4
7
8

Fig. 2 (b)

Supplementary material

Click here to access/download
Supplementary material
Table S1.xlsx
